# In Vivo Analysis of Medial Perforant Path-Evoked Excitation and Inhibition in Dentate Granule Cells

**DOI:** 10.1523/ENEURO.0065-25.2025

**Published:** 2025-12-09

**Authors:** Martin Pofahl, Daniel Müller-Komorowska, Jonas Klussmann, Ilan Lampl, Heinz Beck

**Affiliations:** ^1^Institute of Experimental Epileptology and Cognition Research, University of Bonn, Bonn 53127, Germany; ^2^Department of Neurobiology, Weizmann Institute of Science, Rehovot 7610001, Israel; ^3^Deutsches Zentrum für Neurodegenerative Erkrankungen e.V., Bonn 53127, Germany

**Keywords:** dentate gyrus, granule cells, hippocampus, imaging, in vivo patch clamp, inhibition

## Abstract

Across brain regions and species, the dynamics and balance of excitation and inhibition critically determine neuronal firing. The hippocampal dentate gyrus is a brain area thought to be strongly regulated by inhibition. In vivo, it exhibits remarkably sparse activity, a characteristic proposed to underlie computational tasks like pattern separation. Several populations of interneurons mediate strong feedforward as well as feedback inhibition onto granule cells. However, how the dynamics of inhibition controls granule cell activity in vivo is insufficiently studied. Using two-photon in vivo Ca^2+^ imaging in mice of either sex, we show that sensory stimulation activates only a small number of dentate gyrus granule cells, while inducing widespread inhibition across the remaining granule cell population. Dual-color imaging of both bulk medial perforant path activity and individual granule cell activity allowed us to probe input–output conversion in this pathway. To examine the interplay of MPP-evoked excitation and inhibition at the cellular level, we used in vivo whole-cell patch-clamp recordings, while simultaneously photo-activating MPP inputs. Our findings reveal that MPP-triggered inhibition is fast, significantly larger than excitation, and long-lasting. These results reveal specific properties of inhibition in the dentate gyrus inhibition that are likely crucial for its computational functions, in maintaining sparse activity with a high signal-to-noise ratio.

## Significance Statement

This study investigates the super- and subthreshold computations of dentate gyrus granule cells to an incoming stimulus signal through the medial perforant path the main pathway transferring information from the medial entorhinal cortex layer II into the hippocampus proper. The role of the granule cell network is thought to be crucial for the encoding of new environments and thereby the forming of new memories. Our data directly elucidate the in vivo dynamics of excitation and inhibition in the dentate gyrus using both in vivo imaging and electrophysiology. These findings add to the understanding of the overall sparse code of the dentate gyrus and can be crucial for future studies investigating how hippocampal codes are generated from entorhinal cortex inputs.

## Introduction

The dentate gyrus is a relay structure that conveys multimodal sensory information arriving from the entorhinal cortex to the hippocampus proper. A prevailing concept of dentate gyrus function across species is that it acts as a pattern separator (Leutgeb et al., 2007; Berron et al., 2016; Sakon and Suzuki, 2019), meaning that it is capable of generating dissimilar neuronal representations from similar entorhinal cortex input states (Cayco-Gajic and Silver, 2019). This computational property of the dentate gyrus is supported by expansion recoding, i.e., a mapping of entorhinal cortex information onto a far larger population of granule cells, but also critically requires granule cell activity to be sparse. This is in good agreement with the sparse range of granule cell activity reported in vivo (Pilz et al., 2016; Hainmueller and Bartos, 2018; Pofahl et al., 2021).

The intrinsic and circuit properties of the dentate gyrus are well suited to support sparse coding. In particular, the dentate gyrus constitutes one of the more heavily inhibited regions of the brain. Numerous in vitro studies have shown that afferent activation via the perforant path recruits remarkably strong and fast feedforward and feedback GABAergic inhibition (Ang et al., 2006; Ewell and Jones, 2010; Li et al., 2013; Espinoza et al., 2018; Mircheva et al., 2019; Braganza et al., 2020). Indeed, inhibition is thought to critically contribute to the function of pattern separation (O'Reilly and McClelland, 1994; Stefanelli et al., 2016; Leal and Yassa, 2018; Madar et al., 2019; Braganza et al., 2020). Furthermore, specific activity patterns of somatostatin versus parvalbumin-expressing interneurons in vivo suggest specific roles of inhibitory subcircuits in encoding novelty (Hainmueller et al., 2024) and predicting goal locations (Yuan et al., 2025). Finally, the dentate gyrus is also critical for forms of spatial memory requiring discrimination of subtle differences (Pofahl et al., 2021). Inhibition appears to also be critical for such forms of memory, with plasticity of interneuron recruitment in feedback circuits as a proposed mechanism (Bartos et al., 2001). However, while these studies shed light on the in vivo recruitment of interneurons in the dentate gyrus, it was still unclear how this is translated to dynamic inhibition of granule cells at the single cell and population levels.

Systematic tracing studies have revealed extensive intrahippocampal connectivity contributing to inhibitory networks (Buckmaster and Schwartzkroin, 1995; Sik et al., 1997; Bienkowski et al., 2018; Yen et al., 2022). Because this long-range excitatory as well as inhibitory connectivity may contribute to dynamic inhibition of granule cells, we used in vivo two-photon imaging and patch-clamp recording approaches to assess how perforant path activation evokes dentate gyrus inhibition and how this inhibition impacts the granule cell ensemble as well as its individual cells.

Our results show that mild aversive sensory stimulation leads to recruitment of a small minority of granule cells, with a long-lasting inhibition of the remaining granule cell population. This sensory input drives transmission through the medial perforant path, with a direct correlation between medial perforant path input and the dentate gyrus output. In vivo whole-cell patch-clamp recordings reveal medial perforant path activation generates inhibition that is fast and four times stronger than excitation. The balance between excitation to inhibition is stable across different stimulus intensities and during repetitive stimulation. These properties likely play a role in ensuring sparse granule cell activity across the physiological spectrum of input frequencies.

## Materials and Methods

### Code availability

All code manufactured for this study is available under https://github.com/IEECR/In-vivo-analysis-of-medial-perforant-path-evoked-excitation-and-inhibition-in-dentate-granule-cells.

10.1523/ENEURO.0065-25.2025.d1 Data 1 Download Analysis Code, ZIP file.

10.1523/ENEURO.0065-25.2025.d2 Data 2 Download Analysis Code legend, DOCX file.

### Data availability

All data used in this study is available in a dryad repository: https://doi.org/10.5061/dryad.tdz08kqch.

### Animals and procedures

All animal experiments were conducted in accordance with European (2010/63/EU) and federal law (TierSchG, TierSchVersV) on animal care and use and approved by the county of North-Rhine Westphalia (LANUV AZ 84-02.04.2015.A524, AZ 81-02.04.2019.A216). For imaging experiments, we used 9–12-week-old Thy1-GCaMP6 (6 male, 3 female, GP4.12Dkim/J) mice, expressing GCaMP6s in most excitatory hippocampal neurons (Dana et al., 2014). All Thy1-GCaMP6 mice presented in this study were part of a former study where the histology for virus expression was reported (Pofahl et al., 2021). For all patch-clamp experiments, we used male and female wild type C57/BL6 mice.

### Virus injections and head fixation

Thy1-GCaMP6 mice were anesthetized with a combination of fentanyl/midazolam/medetomidine (0.05/5.0/0.5 mg/kg body weight, i.p.) and head-fixed in a stereotactic frame. Thirty minutes before anesthesia, the animals were given a subcutaneous injection of ketoprofen (5 mg/kg body weight). Eyes were covered with an eye ointment (Bepanthen, Bayer) to prevent drying, and body temperature was maintained at 37°C using a regulated heating plate (TCAT-2LV, Physitemp) and a rectal thermal probe. After removing the head hair and superficial disinfection, the scalp was removed ∼1 cm² around the middle of the skull. The surface was locally anesthetized with a drop of 10% lidocaine, and after 3–5 min, residual soft tissue was removed from the skull bones with a scraper and 3% H_2_O_2_/NaCl solution. After complete drying, the cranial sutures were clearly visible and served as orientation for determining the drilling and injection sites. For virus injection, a hole was carefully drilled through the skull with a dental drill, avoiding excessive heating and injury to the meninges. Any minor bleeding was stopped with a sterile pad. The target site was located as the joint of parietal, interparietal, and occipital skull plates. Subsequently, the tip of a precision syringe (cannula size 34 G) was navigated stereotactically through the burr hole (30° toward vertical sagittal plane, 1.5 mm depth from skull surface) to target the following coordinates: anteroposterior (AP) measured from bregma ∼4.6 mm; lateral (L) specified from midline ∼3 mm; dorsoventral (DV) from surface of the skull ∼4.2 mm. Virus particles [rAAV2/1-CaMKIIa-NES-jRGECO1a (Dana et al., 2016) or rAAV.CamkIIa-hChR2(H134R)-mCherry] were slowly injected (total volume 250 nl, 50 nl/min) in the medial entorhinal cortex. Correct injection site in the medial entorhinal cortex was verified by confined expression of jRGECO1a in the middle molecular layer of the dentate gyrus. To prevent reflux of the injected fluid, the cannula was retained for 5 min at the injection site. For mice used for patching experiments, the skin was sealed using surgical suture. For mice used for imaging, OptiBond (OptiBond 3FL; two-component, 48% filled dental adhesive, bottle kit; Kerr) was applied thinly to the skull to aid adhesion of dental cement. Subsequently, a flat custom-made head post ring was applied with the aid of dental cement (Tetric EvoFlow), the borehole was closed and the surrounding skin adapted with tissue glue. At the end of the surgery, anesthesia was terminated by intraperitoneal injection of antagonists (naloxone/flumazenil/atipamezole, 1.2/0.5/2.5 mg/kg body weight). Postoperative analgesia was carried out over 3 d with once daily ketoprofen (5 mg/kg body weight, s.c.).

### Window implantation procedure

Cranial window surgery was performed to allow imaging of the hippocampal dentate gyrus. Thirty minutes before the induction of anesthesia, the analgesic buprenorphine was administered for analgesia (0.05 mg/kg body weight) and dexamethasone (0.1 mg/20 g body weight) was given to inhibit inflammation. Mice were anesthetized with 3–4% isoflurane in an oxygen/air mixture (25/75%) and then placed in a stereotactic frame. Eyes were covered with an eye ointment (Bepanthen, Bayer) to prevent drying and body temperature was maintained at 37°C using a regulated heating plate (TCAT-2LV, Physitemp) and a rectal thermal probe. The further anesthesia was carried out via a mask with a reduced isoflurane dose of 1–2% at a gas flow of ∼0.5 L/min. A circular craniotomy (Ø, 3 mm) was opened above the right hemisphere hippocampus using a dental drill. Cortical and CA1 tissue was aspirated using a blunted 27-gauge needle until the blood vessels above the dentate gyrus became visible. Even though the aspirated volume was kept to an absolute minimum, we cannot exclude that this type of window surgery might lead to altered dentate gyrus activity. For instance, interneurons projecting from CA1 to dentate gyrus have been described that might normally modulate granule cell activity (Szabo et al., 2017). A custom-made cone-shaped silicon inset (upper diameter, 3 mm; lower diameter, 1.5 mm; length, 2.3 mm; RTV 615, Momentive) attached to by a cover glass (Ø, 5 mm; thickness, 0.17 mm) was inserted and fixed with dental cement. Postoperative care included analgesia by administering buprenorphine twice daily (0.05 mg/kg body weight) and ketoprofen once daily (5 mg/kg body weight, s.c.) on 3 consecutive days after surgery. Animals were carefully monitored twice daily on the following 3 d and recovered from surgery within 24–48 h, showing normal activity and no signs of pain.

### Two-photon calcium imaging

We used a commercially available two-photon microscope (A1 MP, Nikon) equipped with a 25× long-working-distance, water-immersion objective (NA = 1, WD = 4 mm, XLPLN25XSVMP2, Olympus) controlled by NIS-Elements software (Nikon). GCaMP6s was excited at 940 nm using a Ti:Sapphire laser system (∼60 fs laser pulse width; Chameleon Vision-S, Coherent) and a fiber laser system at 1,070 nm (55 fs laser pulse width, Fidelity-2, Coherent) to excite jRGECO1a. Emitted photons were collected using gated GaAsP photomultipliers (H11706-40, Hamamatsu). Movies were recorded using a resonant scanning system at a frame rate of 15 Hz and duration of 20 min per movie.

### Habituation and behavior on the linear track

Imaging experiments were performed in head-fixed awake mice running on a treadmill. Two weeks before the measurements, mice were habituated to the head fixation. Initially mice were placed on the treadmill without fixation for 5 min at a time. Subsequently, mice were head-fixed, but immediately removed if signs of fear or anxiety were observed. These habituation sessions lasted 5 min each and were carried out three times per day, flanked by 5 min of handling. During the following 3–5 d, sessions were extended to 10 min each. The duration of sessions used for experiments was 20 min each.

### Air puff stimulation

During a recording between 36 and 77 air puff stimuli were presented (58 ± 5 stimulations per mouse) on the animals back. The air flow was controlled by a solenoid valve with a pressure of 1 Bar before the valve. The air outlet was a 1 ml pipette tip (Thermo Fisher Scientific). The duration of each air puff was 250 ms. Stimulation only happened when the animal was at rest. The mean probability of delivering an air puff at any given second during resting periods was 0.06. Time intervals between stopping of the animal and the first air puff and interstimulation intervals were randomized (1–50 s) and were applied at random positions on the linear track to prevent the animal from anticipating the stimulation.

### Pupil diameter measurement and analysis

On the linear track, the pupil diameter was measured using a high-speed camera (Basler Pilot, Basler) at a framerate of 100 Hz. To estimate pupil diameter, a circular shape was fitted to the pupil using the LabView NI Vision toolbox (National Instruments), providing a real-time readout. Post hoc, the pupil diameter trace was normalized to its mean. As in a published study (Reimer et al., 2014), frames in which pupil diameters could not be obtained due to blinking or saccades were removed from the trace. The pupil diameter trace was filtered using a Butterworth low-pass filter at a cutoff frequency of 4 Hz. To match the time resolution of the imaging data, the pupil trace was downsampled to 15 Hz. To test whether the pupil constriction after air puffs could serve a classifier for the stimulus, we used ROC analysis. ROC curves were computed by comparing the normalized pupil size in a 1 s time windows pre- versus poststimuli. A series of thresholds was set from the minimum to the maximum number of pupil sizes recorded in each mouse. For each threshold, we calculated the probability that the pupil size was below the threshold in the prestimulus window and in the poststimulus window that defined the false positive rate and the true positive rate, respectively. The ROC curve is produced by plotting the true positive rate against the false positive rate, and the area under the curve (AUC) defines the responsiveness of each cell. To test the significance of responsiveness, we produced shuffled distributions by randomly shuffling the pre- and poststimulus values 1,000 times. For all mice the pupil response resulted in an AUC > 0.7 and above a 95th percentile of its shuffled distribution.

### Individual response analysis and data shuffling

To estimate significant responses of each neuronal or behavioral readout variable for each applied air puff stimulus, we compared the variable with the variance of its baseline and a shuffled distribution. The shuffled distributions were generated by randomly reassigning the times of stimulation to other times when the animal was at rest. Actual stimulation time points were excluded, and every new time point ±1 s could be picked only once. The shuffled data was read out from the shuffled time points, and this procedure was repeated 100 times. The shuffled baseline with 95th percentile for each variable was calculated from the accumulated shuffled data. For each variable, the comparison to its baseline was as follows: The onset of locomotion was considered significant if the speed of the animal exceeded a threshold of 4 cm/s after the stimulus. The pupil response was considered significant if the pupil size after the stimulus exceeded two standard deviations of its size window before the stimulus. The response of a granule cell ensemble was considered significant if the event rate exceeded two standard deviations of its baseline after stimulus. The response of the MPP activity was considered significant for individual stimuli if the Df/F signal after stimulus exceed 2 standard deviations of its baseline.

### Data analysis: two-photon imaging

All analysis on imaging data and treadmill behavior data were conducted in MATLAB using standard toolboxes, open access toolboxes, and custom-written code. To remove motion artifacts, recorded movies were registered using a Lucas–Kanade model (Greenberg and Kerr, 2009). Air puff stimulation or the induced behavioral responses did not induce movement artifacts that were stronger than usual movement artifacts in head fixed imaging data. Individual cell locations and fluorescence traces were identified using a constrained nonnegative matrix factorization-based algorithm, and afterward Ca^2+^ events were identified with a constrained deconvolution algorithm (Pnevmatikakis et al., 2016). The algorithm is dependent on fluorescence changes to identify components and is biased toward active cells. We therefore restricted all analysis to active cells that showed least one Ca^2+^ event with an amplitude 3 standard deviations above noise level in their extracted fluorescence trace. All components were manually inspected and only those that showed shape and size of a granule cell were kept. We binarized individual cell fluorescence traces by converting the onsets of detected Ca^2+^ events to binary activity events. We did not observe any indication of epileptiform activity in Thy1-GCaMP6 (GP4.12Dkim/J) mice, in line with previous work (Steinmetz et al., 2017).

### Analysis of MPP input signals

MPP input bulk signal was analyzed by setting a region of interest in the molecular layer. For that, a threshold of 50% maximum fluorescence was used within the field of view on the average projection of the movie. The bulk fluorescence signal trace was calculated as the average signal of the defined region of interest in each frame. The baseline for the bulk signal was defined as the low-pass filtered signal of the raw trace with a cutoff frequency of 0.01 Hz using a Butterworth filter model. We used a constrained deconvolution algorithm (Pnevmatikakis et al., 2016) to create a proxy for the underlying activity of the bulk signal. This allowed for identification of precise onset times and normalized amplitude values of Ca^2+^ events in MPP input data. To test whether the MPP signal after air puffs could serve a classifier for the stimulus, we used ROC analysis. ROC curves were computed by comparing the normalized Df/F signal in a 1 s time windows pre- versus poststimuli. A series of thresholds was set from the minimum to the maximum number of values recorded in each mouse. For each threshold, we calculated the probability that the Df/F value was below the threshold. For the prestimulus window that defined the false positive rate and for the post stimulus window that defined the true positive rate. The ROC curve is produced by plotting the true positive rate against the false positive rate, and the area under the curve (AUC) defines the responsiveness of the signal. To test the significance of responsiveness, we produced shuffled distributions by randomly shuffling the pre- and poststimulus values 1,000 times. For all mice, the MPP signal resulted in an AUC > 0.85 and above a 95th percentile of its shuffled distribution.

### Air puff responding cells and ROC analysis

To test whether individual granule cell significantly respond to air puffs, we used ROC analysis (Liu et al., 2025). ROC curves were computed by comparing the number of significant calcium transient onsets in a 1 s time windows pre- versus poststimuli. A series of thresholds was set from the minimum to the maximum number of onsets recorded for each granule cell. For each threshold, we calculated the probability that the event value was below the threshold in the prestimulus window and in the poststimulus window that defined the false positive rate and the true positive rate, respectively. The ROC curve is produced by plotting the true positive rate against the false positive rate, and the area under the curve (AUC) defines the responsiveness of the signal. To test the significance of responsiveness, we produced shuffled distributions by randomly shuffling the pre- and poststimulus values 1,000 times. A granule cells was termed a responder if it showed AUC > 0.5 and above a 95th percentile of its shuffled distribution. For the ensemble signals, we used the same approach calculating the sets of thresholds from the averaged and *z*-scored data.

### Fitting response dynamics

To analyze the time courses of the response dynamics, we fitted an exponential rise combined with an exponential decay onto the averaged event probabilities of the responding as well as the nonresponding granule cells. The dynamic function had the following form:

$$f_{\mathrm{dyn}}\;(t)\;=\;A(1-\exp(-t/\tau_{\mathrm{fall}}\;))\;\;\exp(-t/\tau_{\mathrm{rise}}\;)+C,$$

where *A* is the amplitude of the function, *τ*_rise_ is the time constant for the exponential rise, *τ*_fall_ is the time constant of the exponential decay, and *C* is a constant resembling the baseline event probability. For fitting we used the fminsearch MATLAB routine. To estimate the 95% confidence intervals, we used a bootstrapping approach in which we randomly shuffled each data point within its SEM range and repeated the fit to the shuffled data. We repeated this procedure 1,000 times to determine the distributions for the fitted parameters from which we took the 95% confidence intervals. Since the data can be fitted with a wider range of combinations of *A*, *τ*_rise_, and *τ*_fall_, we used the FWHM as a readout to determine the duration of the dynamics.

### Linear mixed model

To test amplitude differences of granule cell ensembles in different behavioral conditions while considering repeated measures and individual mouse contributions, we fitted linear mixed models to the poststimulus amplitudes using the fitlme MATLAB routine. The fitted model was as follows:

response∼condition+(1|animal)+(1|animal:neuron),

where response is the response amplitudes of granule cells, condition is the behavioral condition (air puff with running onset, air puff without running onset, all air puff stimulation, or spontaneous running onset), animal refers to the individual mouse, and animal:neuron refers to the cells nested within individual mice. To test for significance without assuming normally distributed data, we calculated the rank of each cell response and performed a permutation test with 1,000 iterations.

### Correlation analysis

Cross-correlations were calculated using the xcorr MATLAB routine on the average Df/F traces across stimuli of MPP bulk signal and mean granule cell signal. For individual correlations, we used the averaged MPP bulk signal and the individual granule cell signal averaged across trials. The Pearson’s correlation coefficient of these traces was calculated for quantification. For noise correlations, the mean response of each individual cell was subtracted from the individual response of that cell. The same was done for the MPP input trace. The noise correlation was calculated as the Pearson’s correlation using the peak values of these traces across trials.

### In vivo patch-clamp experiments

Three weeks after virus injection, mice were anesthetized with ketamin (0.1 ml ketamin, 0.075 ml xylazine, 0.225 ml Saline) and the analgesic buprenorphine was administered for analgesia (0.05 mg/kg body weight). Eyes were covered with an eye ointment (Bepanthen, Bayer) to prevent drying and body temperature was maintained at 37°C using a regulated heating plate (TCAT-2LV, Physitemp) and a rectal thermal probe. After removal of the head hair and superficial disinfection, the scalp was removed ∼1 cm² around the middle of the skull. The surface was locally anesthetized with a drop of 10% lidocaine, and after 3–5 min residual soft tissue was removed from the skull bones with a scraper and 3% H_2_O_2_/NaCl solution. After complete drying, the cranial sutures were clearly visible and served as orientation for the determination of the drilling and injection sites. A custom-made headpost was fixed on the skull using UV curing glue (4305, Loctite) and the animal was head fixed. A tube was inserted into the animal's trachea to control breathing, monitor oxygen level, and induce the anesthesia with 1–2% isoflurane throughout the experiment. A multimode light fiber was implanted into MEC through the craniotomy that was drilled during virus injection under an angle of 15° with respect to the anteroposterior axis. Above the hippocampus the skull was carefully opened using a dental drill. The dura was carefully removed without injuring the underlying cortex tissue. The blind patching followed a formerly described protocol (Okun and Lampl, 2008; Cohen-Kashi Malina et al., 2016). Briefly, a patch pipette was vertically inserted into the cortex and carefully lowered to the depth of the DG granule cell layer. The intracellular solution for current-clamp experiments contained the following (in mM): 136 K-gluconate, 5 NaCl, 10 KCl, 10 HEPES, 1 MgATP, 0.3 NaGTP, 10 phosphocreatine, 2 QX314, biocytin. The approach to a putative granule cell was identified monitoring resistance response at the pipette tip. After successful giga-seal and cell opening, the cell was measured in current clamp. For a unique identification of cells, only one cell per session was measured. During experiments MEC cell somata were stimulated with 473 nm light administered from a diode laser through the implanted light fiber.

Inhibitory and excitatory conductance were calculated following a formerly described procedure (Priebe and Ferster, 2005). Briefly, a simplified membrane equation was utilized:

$$I_{\mathrm{inj}}=C\frac{dV}{dt}+G_{r}(V_{t}-E_{r})+G_{e}(V_{t}-V_{e})+G_{i}(V_{t}-V_{i}),$$

where *I*_inj_ is the clamped current, *C* is the membrane capacitance, *V* is the measured voltage, *G_r_* is the leak conductance, *E_r_* is the resting potential, and *V_e_* and *V_i_* are the summarized reversal potentials for all excitatory and inhibitory channels, respectively. *G_e_* and *G_i_* are the excitatory and inhibitory conductance, respectively. The resting membrane potential was measured in resting condition of the cell. The reversal potentials were assumed as constant and were defined by the in-solution composition. The leak conductance *G_r_* was estimated using the voltage step size after current injection at steady state:

$$G_{r}=\Delta I/\Delta V,$$

where the final conductance was taken as the average of all current voltage combinations during one measurement. The membrane capacitance *C* was estimated by fitting time course of the membrane charging after current injection and estimating the charging time constant *τ*. The capacitance is then given by the following:

$$C=\tau \;G_{r}.$$

To correct for nonideal patching conditions the constants were further fitted by injecting different current values so that the resulting passive properties of the cell were matched. Conductances were measured by measuring the voltage response to MPP stimulation at four different clamped current values. Each measurement delivered a linear solution for the conductances, so in total four linear solutions. The final values for both conductances were set as the center of mass of all intersections of all four solutions. To test the found solutions, theoretical voltage traces were reconstructed that used the estimated values for the conductances. To estimate the goodness of fit, the mean squared error, the *R*^2^ value, and the Pearson’s correlation between the input voltage traces and the reconstructed traces were calculated for time windows of 1 s after each stimulus.

Post hoc, the biocytin filled cell was stained with streptavidin 488 and recovered in fixed tissue. Cell nuclei were stained with DAPI allowing the identification of the granule cell layer. A measured cell was included only if it was found within the granule cell layer and morphologically identified as granule cell (21/35 of patched cells). Experiments were included only if viral expression could be confirmed within the medial molecular layer of DG (9/21 of successful patching sessions).

### In vitro patch-clamp experiments

Isoflurane anesthesia was used to rapidly decapitate adult male and female mice 10–14 weeks old expressing ChR2 in the MEC (see viral injection procedures). Dissected brains were transferred to carbogenated artificial cerebrospinal fluid (ACSF) with sucrose (ice cold; in mM: 60 NaCl, 100 sucrose, 2.5 KCL, 1.25 NaH_2_PO_4_, 26 NaHCO_3_, 1 CaCl_2_, 5 MgCl_2_, 20 glucose; from Sigma-Aldrich). Then, 300-μm-thick coronal slices were transferred to the same ACSF with sucrose at 37°C for 20 min. Slices were then kept and sliced in ACSF without sucrose (in mM: 125 NaCl, 3.5 KCL, 1.25 104 NaH_2_PO_4_, 26 NaHCO_3_, 2 CaCl_2_, 2 MgCl_2_, 20 glucose; from Sigma-Aldrich). The intracellular solution for voltage-clamp experiments contained the following (in mM): 120 Cs methanesulfonate, 0.5 MgCl_2_, 5 2-(4-(2-hydroxyethyl)-1-piperazinyl)-ethansulfonsäure (HEPES), 5 ethyleneglycol-bis(aminoethylether)-*N*,*N*,*N*′,*N*′-tetraacetic acid (EGTA), 5 adenosine 5′-triphosphate disodium salt (Na_2_-ATP), 5 *N*-(2,6-dimethylphenylcarbamoylmethyl)triethylammonium chloride (QX 314 Cl−); from Sigma-Aldrich. Granule cells were patched at room temperature central in the upper blade of dorsal dentate gyrus, to target mature granule cells comparable to those targeted in the in vivo patching and imaging experiments. To isolate monosynaptic responses, we used ACSF with 1 μM tetrodotoxin (TTX, Tocris), 200 μM 4-aminopyridine (4-AP, 112 Sigma-Aldrich). To isolate excitatory conductances, we used 10 μM gabazine (SR 95531 hydrobromide; Tocris). Patch-clamp experiments were performed with a MultiClamp 700B and digitized on an Axon Digidata 1550A. Light stimulation was performed with an Omicron LuxX 473 nm laser attached to a light fiber submerged in the ACSF. Light stimuli were 10 ms long. To isolate excitatory and inhibitory conductances, we assumed a chloride reversal of −80 mV and a cation reversal potential of 0 mV. Furthermore, we used gabazine to isolate the excitatory conductance. The excitatory conductance we report was calculated with gabazine washed in at a corrected holding potential (liquid junction potential: −9 mV) of −80 mV. The inhibitory conductance we report was calculated at a corrected holding potential of 0 mV and subtracting the response recorded with gabazine from the baseline response under ACSF. Recordings were performed at room temperature.

## Results

### Sensory stimulation leads to an activation of a small subset of granule cells and widespread inhibition of the remaining population

**Figure 1.**
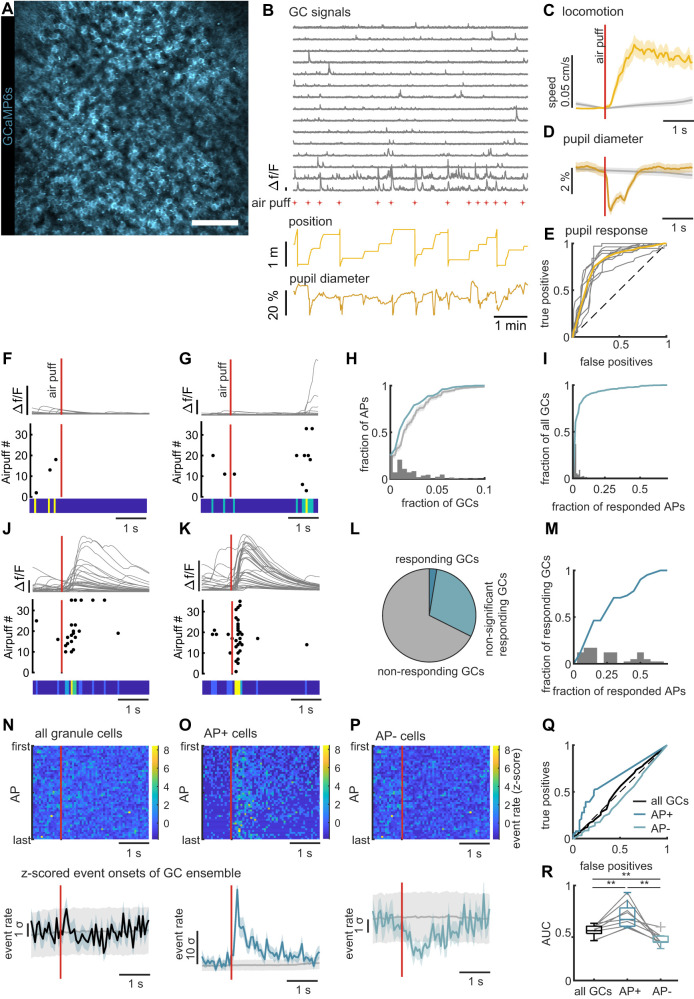
Air puff stimulation triggers sparse excitation and widespread inhibition in the granule cell network. ***A***, Example field of view of granule cell imaging in dentate gyrus. Scale bar, 100 µm. Extended Data Figure 1-1*A* shows the number of active granule cells per FOV. ***B***, Data of example granule cell recording. Gray traces: 15 representative GCaMP6s fluorescence signal traces of individual granule cells. Red asterisks, Times of air puff stimulation. Light yellow trace, Mouse position on the running belt. Dark yellow trace, Pupil diameter. Extended Data Figure 1-1*B–D* shows the number and timing of the puff stimuli. ***C***, Running onsets after air puff stimulation (yellow line with SEM). Running speed averaged over all stimuli and mice (*n* = 519 stimuli in 9 mice). Gray line represents shuffled data with 95th percentiles. ***D***, Pupil constriction after air puff stimulation (yellow line with SEM). Pupil diameter averaged over all stimuli and mice (*n* = 519 stimuli in 9 mice). The gray line represents shuffled data with 95th percentiles. Extended Data Figure 1-1*E* shows the percentage of significant pupil constrictions for each animal. Extended Data Figure 1-1*F–I* shows neuronal and behavioral readouts for stimuli that did not cause significant pupil constriction. ***E***, ROC analysis for the pupil constriction following air puff stimulation. Gray lines represent the response curves from individual animals; yellow line represents the response curve of the pooled data (*n* = 9 mice). ***F***, ***G***, Examples activity profiles of two granule cells around the air puff stimulation. Top panels: Denoised Ca^2+^ activity (gray traces) aligned to stimulus times (red bars). Middle panels, Raster plots of the Ca^2+^ event onsets after individual air puff stimulations. Bottom panels, Heat map reflecting the average of Ca^2+^ event onsets. ***H***, Histogram showing the number of activated granule cells per air puff (gray bars). The blue line represents the cumulative distribution of the same data. The gray line represents the distribution of shuffled data with 95th percentile. The mean of responses after air puffs is significantly lower than for the shuffled data (permutation test, *n* = 1,000 iterations, *p* = 0.003). ***I***, Histogram showing the number of responded air puffs per granule cell (gray bars) for all granule cells that were active at least once following an air puff. The blue line represents the cumulative distribution of the same data. ***J***, ***K***, Examples of two air puff responding granule cells. Top panels, Denoised Ca^2+^ activity (gray traces) aligned to stimulus times (red bars). Middle panels, Raster plots of the Ca^2+^ event onsets after individual air puff stimulations. Bottom panels, Heat map reflecting the average of Ca^2+^ event onsets. Extended Data Figure 1-1*J* shows the ROC curves for individual granule cells. ***L***, Fraction of significantly responding granule cells (2.6%, dark blue), nonsignificantly responding granule cells (29.9%, light blue) and never responding granule cells (67.5%, gray) of all granule cells in the dataset. Extended Data Figure 1-1*K*,*L* shows number and percentage of significant responding granule cells per animal. ***M***, Histogram showing the number of responded air puffs per granule cell (gray bars) for significantly responding granule cells. The blue line represents the cumulative distribution of the same data. ***N***, Top panel, Averaged activity of all granule cells in a 4 s window around every air puff across session (*n* = 1,583 cells from 9 mice). Event onsets per frame were averaged over cells and then *z*-scored. To match different numbers of stimuli, the responses were resampled to 50 stimuli. The color bar indicates the sigma values of the *z*-scored signal. The red line denotes the time of the air puff presentation. Bottom panel, *Z*-scored Ca^2+^ event onset probability of all granule cells (black line with SEM). Data is averaged over all cells and stimuli and *z*-scored with respect to baseline. The gray line represents shuffled data with 95th percentiles. ***O***, Same as ***N*** but only for significantly responding AP+ granule cells (*n* = 38 cells from 9 mice). ***P***, Same as ***N*** for the AP− cells (all granule cells except the significantly responding ones, *n* = 1,545 cells from 9 mice). ***Q***, ROC analysis curves for the mean signals of all granule cells. For all cells (black line) the difference to 0.5 is not significant. For AP+ cells (dark blue line), the AUC is closer to 1 and significant against shuffle. For the AP− cells (light blue line), the curve is slightly less than 0.5 and significant against shuffle. Extended Data Figure 1-1*N–P* shows the ROC curves for individual animals. ***R***, Comparison of AUC for all granule cells (black), AP+ cells (dark blue) and AP− cells (light blue) for different mice. Gray lines denote individual animals. Repeated-measures ANOVA (*n* = 9 mice, *F* = 22, *p* < 0.001), Bonferroni’s correction for pairwise comparisons all *p* < 0.01). Extended Data Figure 1-2 shows the comparison for different responder different responder definitions. Panels ***L***, ***O***, ***P***, and ***Q*** are reproduced for these different definitions.

We imaged the activity of 1,583 active granule cells [176 ± 57 active cells per field of view (FOV)] in nine mice expressing GCaMP6s under the Thy1 promoter (GP4.12Dkim/J; Dana et al., 2014; Extended Data Fig. 1-1*A*). Two-photon imaging in head-fixed mice was carried out while the mice ran on a linear track (Fig. 1*A*,*B*). To prevent confounding factors of landmarks or spatially defined food rewards, the mice ran in darkness without any reward on a running belt that was cue enriched but without separate zones or distinct landmarks. We used a constrained nonnegative matrix factorization-based algorithm to identify active granule cells in each FOV (Pnevmatikakis et al., 2016). All analysis in this study is restricted to active granule cells and the onsets of significant Ca^2+^ transients are used as a readout of cell activity. To examine the activity responses of granule cells to a physiological input signal, we used a mild air puff administered to the back, since air puff stimulation has been shown to cause reliable activation of hippocampal neurons (Barth et al., 2023). Because the activity profile of dentate granule cells in head-fixed mice can vary significantly with locomotor state, we restricted all stimulation times to periods of immobility (Fig. 1*C*; Danielson et al., 2016; Pofahl et al., 2021). During a 20-min-long session 36–77 air puff stimuli were presented (Extended Data Fig. 1-1*B*, 519 stimulations in total, 58 ± 5 stimulations per mouse). To prevent the animal from anticipating the stimulation, time intervals between stopping of the animal and the first air puff and interstimulation intervals were randomized (1–50 s; Extended Data Fig. 1-1*C*,*D*) and were applied at random positions on the linear track. We used pupil dynamics as a readout of successful stimulation since air puffs can trigger responses in pupil size (Fig. 1*D*). ROC analysis revealed that rapid pupil constrictions following the stimulus can serve a classifier for air puff stimulation which was significant for every individual mouse (Fig. 1*E*). To define a successful stimulation, we checked whether the pupil constriction in each individual trial exceeded a 2-sigma threshold compared with baseline, which was the case in 92 ± 2% of all stimulus presentations and reliable across mice (Fig. 1*D*, Extended Data Fig. 1-1*E*, *n* = 519 stimuli). For trials without pupil constriction, we found indeed less evidence of the responses we found in later parts of this study (Extended Data Fig. 1-1*F–I*). The observed rapid pupil constrictions are distinct from slower pupil dilations observed after changes in locomotor state (Pofahl et al., 2021).

10.1523/ENEURO.0065-25.2025.f1-1 Figure 1.1 Figure 1-1: **Imaging dentate gyrus granule cell responses during an air puff stimulation paradigm.** **A**, Numbers of identified active granule cells for individual mice. **B**, Numbers of applied air puff stimuli per mouse **C**, Time intervals between stopping of an animal and the first air puff stimulation **D**, Time intervals between airpuffs **E,** Fraction of air puffs that triggered a significant pupil response for individual mice. **F**, Mean of granule cell activity after air puffs that did not trigger significant pupil dilation for all granule cells except significant responders. **G**, Mean of granule cell activity after air puffs that did not trigger significant pupil dilation for significantly responding granule cells. **H**, Mean of running speed after air puffs that did not trigger significant pupil dilation. **I**, Mean pupil dynamics after air puffs that did not trigger significant pupil dilation. **J**, ROC curves for all significantly responding granule cells **K**, Numbers of significantly responding granule cells for individual mice **L**, Fractions of significantly responding granule cells for individual mice **M**, Correlation of response probability of individual responding granule cells to their overall activity rate (n = 38 cell from 9 mice, r = 0.68, p < 0.001) **N**, ROC curves for the mean activity rate of all granule cells for individual mice (grey lines) and the pooled data set (Black line). **O**, like K for the mean of significantly responding granule cells **P**, like K for the mean of all granule cells except the responding granule cells **Q**, Correlation of the AUC derived from the mean signal of significantly responding granule cells from individual mice against the absolute number of responders in each FOV (n = 9 mice, r = 0.79, p = 0.01) **R**, (Non-) correlation of the AUC derived from the mean signal of significantly responding granule cells from individual mice against the absolute number of responders in each FOV (n = 9 mice, r = -0.2, p = 0.61). Download Figure 1.1, TIF file.

**Figure 2.**
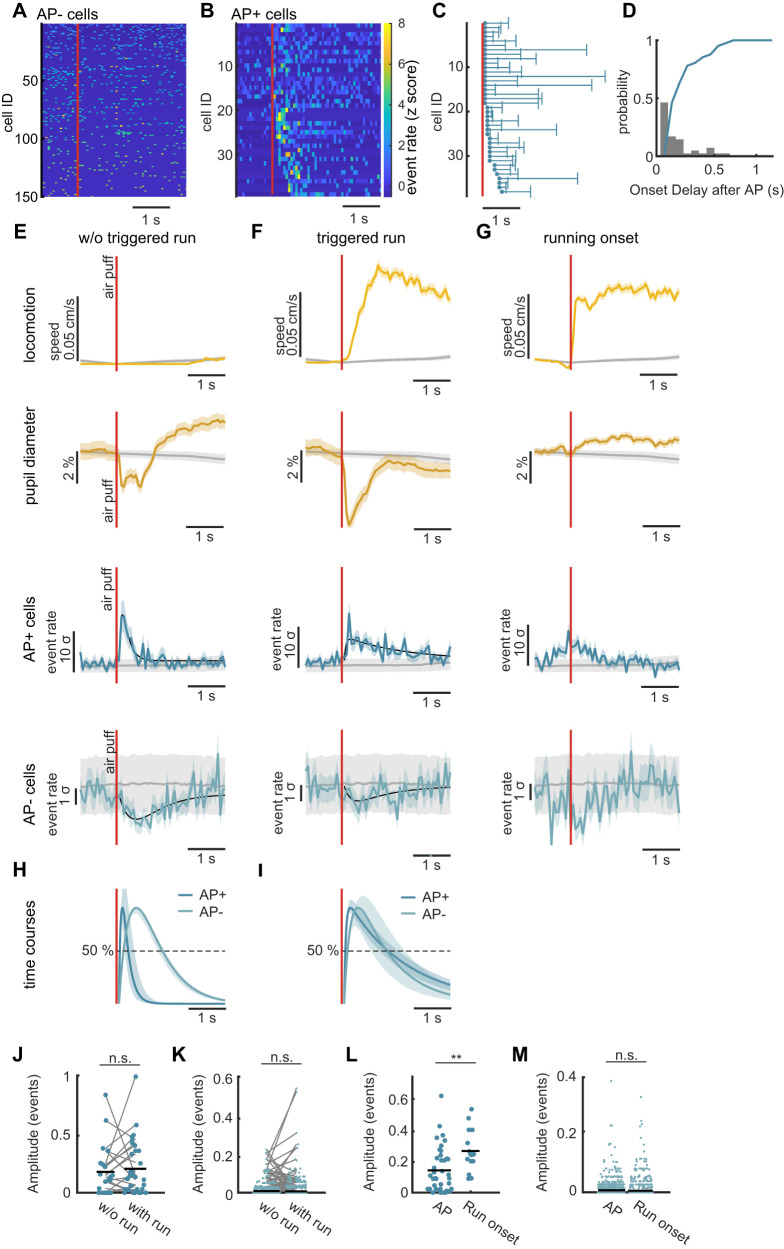
Time course of activation versus inhibition of granule cells. ***A***, *Z*-scored heatmaps of 150 nonresponding AP− granule cells from one example mouse. Color bar similar to panel ***B*** for *z*-score values. ***B***, *Z*-scored heatmaps of all air puff responding AP+ granule cells of all mice (*n* = 38 cells from 9 mice). Color bar denoting the *z*-score value. ***C***, Time courses of AP+ cells shown in ***B***. Points represent the response onset of each cell and the whisker the FWHM of the activation of the respective cell. ***D***, Histogram showing the number of AP+ cells with a specific response onset delay (gray bars). The blue line represents the cumulative distribution of the same data. ***E***, For air puffs without triggered running onset: Top yellow panel, Mean running speed after air puff. Bottom yellow panel, Mean pupil dynamics. Top blue panel, *Z*-scored Ca^2+^-event onsets of AP+ cells. Data is averaged over all cells and stimuli and *z*-scored with respect to baseline. Bottom blue panel, *Z*-scored Ca^2+^ event onsets of AP− cells. Shaded area in all panels denotes SEM and the gray line represents shuffled data with 95th percentiles. Extended Data Figure 2-1 shows the relation between granule cell activation and triggered running initiation for individual animals. ***F***, Like ***E*** for air puff stimuli that triggered running onsets. ***G***, Like ***E*** for spontaneous running onsets. ***H***, Normalized time courses for the mean activity of AP+ cells (dark blue line) and of AP− granule cells (light blue line) as fitted from the granule cell activity in ***E***. For comparison the sign of the inhibited granule cell activity signals was inverted. Filled area depicts the 95% confidence interval of each fit after bootstrapping. ***I***, Same as ***H*** with data from ***F*** for air puff stimuli that triggered running onsets. ***J***, Swarm chart of response amplitudes of AP+ cells after air puff stimuli that caused running initiation versus air puffs that did not cause running initiation; black lines depict the mean values. Permutation test on the ranks of cells, *p* = 0.52, *n* = 46 cells. ***K***, Same as ***J*** for AP− cells. Permutation test on the ranks of cells, *p* = 0.49, *n* = 1,936 cells. ***L***, Swarm chart of response amplitudes of AP+ cells after air puff stimulation and for spontaneous running onsets without air puff stimulation; black lines depict the mean values. Permutation test on the ranks of cells, *p* = 0.001, *n* = 52 cells. ***M***, Same as ***L*** for AP− cells. Permutation test on the ranks of cells, *p* = 0.11, *n* = 2,954 cells.

10.1523/ENEURO.0065-25.2025.f1-2 Figure 1.2 **Effects are robust for different responder definitions** **A**, Venn diagram illustrating the granule cells identified for the AP+ group using different responder definitions. ROC analysis (purple), shuffling approach with 95^th^ percentile (blue), mutual information score (orange), and glm based identification (yellow). **B**, Like A with an additional threshold of 5% of responded stimuli per cell. **C**, Analogous to panel 1L for different responder definitions **D**, Analogous to panel 1O for different responder definitions **E**, Analogous to panel 1P for different responder definitions **F,** Analogous to panel 1Q for different responder definitions. Download Figure 1-2, TIF file.

We first asked if there is an observable neuronal response to the air puff stimulation in dentate gyrus. Granule cells show generally low activity levels, which can individually vary over a wide range (Pilz et al., 2016; Hainmueller and Bartos, 2018; Pofahl et al., 2021). Indeed, we found the activity of granule cells around the air puff stimulation to be very sparse (Fig. 1*F*,*G*). We therefore first quantified how many granule cells had at least a single detected calcium transient onset in a 1-s-long response window after a given air puff (Fig. 1*H*). In line with the generally sparse activity, this analysis showed that on average 2% of granule cells were active following each air puff. Further, we found that 23% of air puffs were not followed by any granule cell activation and that no air puff was followed by >10% active cells of the imaged granule cell population. The comparison to a shuffled distribution showed that the mean recruitment of granule cells following the air puff was even less than the average number of active cells during other moments of immobility (Fig. 1*H*, gray line, permutation test of mean, *p* = 0.003). Next, we asked how reliably those granule cells that were active following the stimulus were activated. We found that 32% of granule cells were active following a stimulus at least once and 18% were active exactly once (Fig. 1*I*). However, this distribution had a long tail saturating at 67% of responded stimuli. This suggested that there is a small subset of granule cells that is more reliably triggered by the air puff stimulation.

To test whether individual granule cells significantly respond to air puffs, we used ROC analysis (Liu et al., 2025) to compare the activity of individual granule cells after the stimulation to the baseline activity. If this difference could classify the occurrence of an air puff with an area under the curve (AUC) > 0.5 and above a 95th percentile chance level, the granule cell was considered a significant responder (Extended Data Fig. 1-1*J* and Materials and Methods). Using this quantitative responder definition (Fig. 1*J*,*K*), we found in total 38/1,583 responders (Fig. 1*L*), corresponding to 2.6 ± 0.6% of granule cells across mice (Extended Data Fig. 1-1*K*,*L*). We termed the significantly responding granule cells AP+ cells and summarized all other granule cells as AP− cells. Within the AP+ set, half of the cells responded to <25% of stimuli, with a small group of cells responding to ∼50% of the stimuli (Fig. 1*M*, *n* = 38 cells from 9 mice). This demonstrates that even in AP+ cell set, responses are quite unreliable across stimulations. These differences in air puff responsiveness are correlated to the overall cell activity during a recording (Extended Data Fig. 1-1*M*, *r* = 0.66, *p* < 0.001, *n* = 38 cells), suggesting that an overall excitability determines how reliable a cell can follow incoming stimuli. Of note, the exact definition of the AP+ granule cells did not change any of the results, as five different definitions of responders were tried for the analysis which all led to a high overlap of identified cells and comparable results (Extended Data Fig. 1-2). The ROC analysis was chosen since it was the most conservative definition and contained only cells that were also identified by other definitions (Extended Data Fig. 1-2*A*,*B*).

Consistent with the sparse air puff responses, quantifying the responses to a given air puff averaging all granule cells across mice did not show a clear peak following any air puff stimulation (Fig. 1*N*, top panel). Accordingly, the grand average of this signal was within the 95th percentile of the shuffled signal (Fig. 1*N*, bottom panel). When restricting this analysis only to the AP+ cells, we expectedly identified a clear peak after the air puffs (Fig. 1*O*, top panel) also clearly visible in the grand average (Fig. 1*O*, bottom panel). We then looked at the activity of the complementary AP− cells. Even though the baseline levels of activity in these granule cells were low, the averaged event rates of these cells dropped even further after air puff stimulation (Fig. 1*P*, top panel). This was reflected in a significant drop of activity after air puff stimulation compared with a shuffled distribution (Fig. 1*P*, bottom panel, *n* = 1,545 cells). These results suggest a widespread inhibition of the AP− cell set, triggered by the air puff stimulation.

To analyze whether the ensemble signal in any of these group definitions could serve as a classifier for the air puff stimuli, we performed ROC analysis on the average signals (Fig. 1*Q*). The AUC derived from average signal from all granule cells was ∼0.5 in all animals and could not decode the air puff times better than shuffled data (Fig. 1*Q*; individual mice in Extended Data Fig. 1-1*N*). The AP+ cells could decode the air puff times in all mice better than the shuffled data (Fig. 1*Q*; individual mice in Extended Data Fig. 1-1*O*). Still, the AUC was close to 0.5 in some animals that had a small number of significant responders. Overall, the performance of this analysis was correlated to the absolute number of identified AP+ cells (Extended Data Fig. 1-1*Q*) but not to differences in the relative number of responders (Extended Data Fig. 1-1*R*). For the AP− cells, we found AUCs significantly lower than 0.5 in 8/9 animals (Extended Data Fig. 1-1*P*) and a similar phenomenon in the average of all mice (Fig. 1*Q*). Statistical analysis showed that AP+ cells had a significantly higher AUC, while the AP− cells had a significantly lower AUC compared with all granule cells [Fig. 1*R*; repeated-measures ANOVA (*n* = 9, *F* = 22, *p* < 0.001), Bonferroni’s correction for pairwise comparisons all *p* < 0.01]. This shows that decoding of the air puff is not possible with the average activity of all granule cells, but the presence of an air puff can be classified using only the responding AP+ cell population, as well as to a lesser extent the complementary AP− cell population.

### Excitation and inhibition of granule cells follow different time courses

Next, we asked if the time courses of the excitation and the inhibition of granule cells following the air puff stimulation differ from each other. To this end, we analyzed the activity of individual granule cells averaged over all stimulations. AP− granule cells not belonging to the responder group showed a slight but visible decrease of activity (150 randomly drawn examples in Fig. 2*A*). The significantly responding AP+ cells, on the other hand, showed clear and sharper responses (Fig. 2*B*). We analyzed time course of the responses by fitting the averaged responses of each individual cell with a double exponential function (see Materials and Methods). We found that some responses were sharp, while others seem to maintain an elevated activity level after their response onset (Fig. 2*B*,*C*). We also found that the response onsets after stimuli varied across cells (Fig. 2*C*,*D*). While ∼50% of granule cells respond faster than 150 ms (corresponding to two frames in our recording), we found that onset delays can be as late as 500 ms.

10.1523/ENEURO.0065-25.2025.f2-1 Figure 2-1 **Granule cell responses are not correlated with triggered running initiationA**, Bar graph denoting the fraction of air puffs that triggered running (solid yellow), a granule cell ensemble response (solid blue), both (blue and yellow), or neither (grey) for individual mice. **B**, Scatter plot showing each individual responding granule cell in each animal the fraction of responses that were combined with a triggered running initiation. **C**, Histogram counting the cells with a specific fraction of responses that were combined with a triggered running initiation **D**, same as B but only for mice that showed granule cells responses that were combined with and without a triggered running initiation. **E**, same as C but only for mice that showed granule cells responses that were combined with and without a triggered running initiation **F**, Intercepts of individual animals from the test presented in panel 2J. The overall intraclass correlation is 0.19. **G**, Intercepts of individual animals from the test presented in panel 2K. The overall intraclass correlation is 0.004.**H**, Intercepts of individual animals from the test presented in panel 2L. The overall intraclass correlation is 0.2. **I**, Intercepts of individual animals from the test presented in panel 2M. The overall intraclass correlation is 0.04. Download Figure 2-1, TIF file.

Given these differences in response dynamics, we wondered if the activity of individual granule cells or the cell ensemble is influenced by the onset of locomotion since the input–output dynamics of the dentate differ between locomotion and rest (Pofahl et al., 2021). Therefore, we subdivided the stimulation episodes into those which were associated with no running response (Fig. 2*E*, air puff without triggered run) and those that triggered a running onset (Fig. 2*F*, air puff with triggered run). Air puff stimuli triggered running onsets in 48 ± 9% of stimulations. At the same time 55 ± 10% of air puffs led to a significant response in the ensemble signal of AP+ granule cells, where a significant response was defined as an amplitude of two sigma above the baseline level. There was no systematic correlation between triggered running onsets and significant responses of the AP+ ensemble signal (Extended Data Fig. 2-1*A*). For individual AP+ cells, we found that some cells seemed to respond preferentially in scenarios with or without a running onset but did not observe a general trend for one preference (Extended Data Fig. 2-1*B–E*). We observed differences in pupil diameter between conditions, at later stages >1 s after the air puff stimulation. This can be explained by a general correlation between running speed and pupil expansion leading to more dilated pupils at rest than during locomotion (Fig. 2*G*; Pofahl et al., 2021).

We then looked systematically at the activity of the AP+ and AP− groups in response to stimulation with and without locomotion onsets. For stimuli without triggered running initiation, AP+ cells show a sharp positive response, while AP− cells displayed an average reduction of activity, as previously observed for the mean of all air puff stimulations (Fig. 2*E*). For the air puffs that triggered running onsets, we found slowly decaying responses in the AP+ cells (Fig. 2, compare *F*, *E*). For the AP− cells, the negative peak of the response did not exceed the 95th percentile of the shuffled distribution but still showed similar dynamics as in the previous analysis (Fig. 2*F*). We described response dynamics by fitting a double exponential function (Fig. 2*E*,*F* black lines, and see Materials and Methods) and compared the time courses of the normalized inhibitory and excitatory responses. Following air puff stimulations without a run onset, the activation of AP+ cells displayed a much faster time course than the deactivation of the AP− cells (Fig. 2*H*; FWHM 262 ± 30 ms for AP+ cells versus 1,183 ± 60 ms for AP− cells. Values with 95% confidence intervals after boot strapping). For the stimuli that triggered locomotion, we found that the decay of the AP+ cell response was indeed longer than in the other scenario while the deactivation of the AP− cells followed a similar time course (Fig. 2*I*; FWHM 1,457 ± 90 ms for AP+ cells versus 1,222 ± 63 ms for AP− cells. Values with 95% confidence intervals after boot strapping).

We also compared the response amplitude of AP+ and AP− cells with respect to whether the air puff stimulation triggered locomotion, by fitting a linear mixed effects model to each group of cells, considering the repeated measures of cells and mice-wise contributions. In this test we included only mice that showed behaviorally both types of responses. Using this test design, we did not find a difference in response amplitude for the AP+ cells (Fig. 2*J*; permutation test on the ranks of cells *p* = 0.52, *n* = 46), nor for the AP− cells (Fig. 2*K*; permutation test on the ranks of cells *p* = 0.49, *n* = 1,936). An intraclass correlation < 0.3 (0.19 and 0.004 for AP+ and AP− cells, respectively) showed that the test result was not driven by individual animals (Extended Data Fig. 2-1*F*,*G*).

To control whether running onsets alone would give rise to similar response dynamics or how the observed dynamics could be influenced by the locomotion onset, we analyzed a set of sessions without air puff stimulation recorded from the same animals and used the running onsets as stimulation times (Fig. 2*G*). Using the same criteria as for the air puff response, we found that across animals 1.5 ± 0.4% of active granule cells responded to the running onset, which is lower than that observed to the air puff response (2.6%). However, the dynamics did not show the sharp onset we observed for air puff stimulation. We compared the amplitudes of responsive and nonresponsive granule cells between spontaneous running onsets and post air puff stimulation amplitudes. For responsive cells we found a significant difference between air puff and spontaneous running onsets (Fig. 2*L*; permutation test on the ranks of cells *p* = 0.001, *n* = 52), where the mean amplitudes of individual cells were higher for the cells that responded to running onsets. We did not find a significant difference for the amplitudes of nonresponding cells (Fig. 2*M*; permutation test on the ranks of cells *p* = 0.11, *n* = 2,954). Again, an intraclass correlations were <0.3 (0.2 and 0.04 for responsive and nonresponsive granule cells, respectively), which showed that the test result was not driven by individual animals (Extended Data Fig. 2-1*H*,*I*). Taken together, the onset of locomotion cannot explain the response dynamics for the AP+ or the AP− cells that we observed but could act as a modulating factor explaining the differences we found between the stimulation with and without triggered locomotion onsets.

### Correlation of MPP input activity with granule cell responses

**Figure 3.**
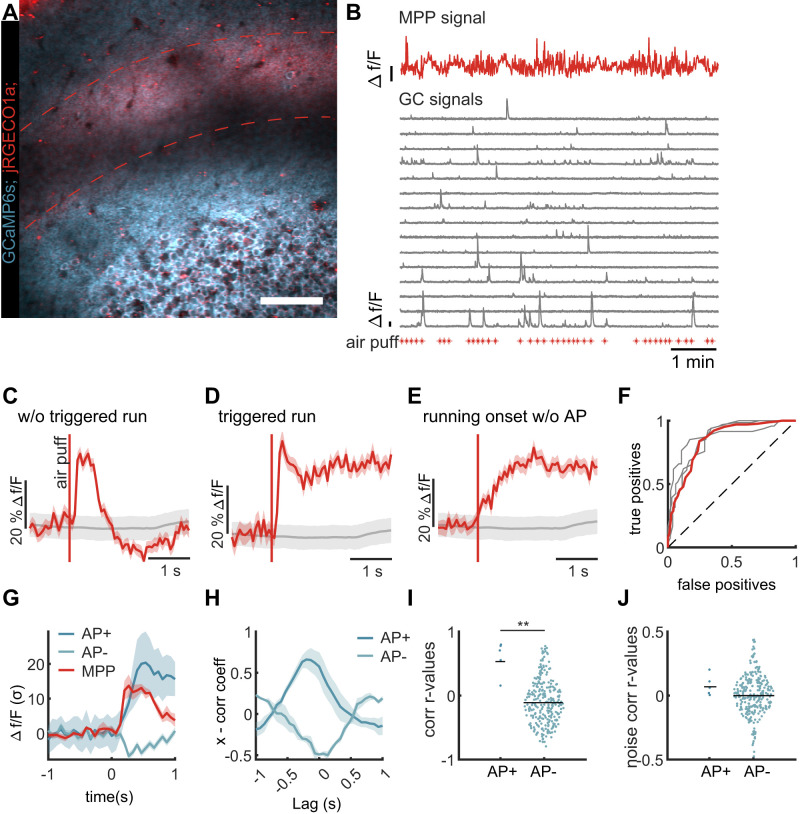
Sensory-triggered MPP activation correlates to excitation and inhibition of dentate granule cells. ***A***, Example field of view of dual-color recording of MPP fiber bundle (red, between dashed lines, jRGECO1a) and granule cells (blue, GCaMP6s) in dentate gyrus. Scale bar, 100 µm. ***B***, Example of dual-color MPP and granule cell recording. Red trace, Bulk fluorescence signal of MPP fiber bundle. Gray traces, 15 representative GCaMP6s fluorescence signal traces of individual granule cells. Red asterisks, Times of air puff stimulation. ***C***, Mean MPP fluorescence signal after air puff stimulation when no locomotion was triggered. DF/F signal averaged over all stimuli and mice with SEM (*n* = 3 mice). The gray line represents shuffled data with 95th percentiles. ***D***, Mean MPP fluorescence signal after air puff stimulation when locomotion was triggered. DF/F signal averaged over all stimuli and mice with SEM (*n* = 3 mice). The gray line represents shuffled data with 95th percentiles. ***E***, Mean MPP fluorescence at times of spontaneous running onsets. DF/F signal averaged over all onsets and mice with SEM (*n* = 3 mice). The gray line represents shuffled data with 95th percentiles. ***F***, ROC analysis for the MPP bulk signal following air puff stimulation. Gray lines represent the response curves from individual animals; the red line represents the response curve of the pooled data (*n* = 3 mice). ***G***, *Z*-scored DF/F signals of the MPP bulk signal (red line), the responding AP+ granule cells (dark blue line), and the other AP− granule cells (light blue line). All traces with SEM. ***H***, Cross-correlation of the DF/F traces from ***G***. Between MPP and AP+ cells (dark blue) and the AP− cells (light blue) with SEM (*n* = 3 mice). ***I***, Correlation coefficients between MPP response amplitudes and responses of individual AP+ cells (dark blue dots, *n* = 5 cells) and for AP− cells (light blue dots, *n* = 263 cells), Mann–Whitney *U* test *p* < 0.001, *n* = 5 versus 263 cells for AP+ and AP−, respectively. Subsampling with size matched groups median *p* = 0.016, 80% of *p* values < 0.05, 1,000 iterations. ***J***, Noise correlation coefficients between mean corrected MPP response amplitudes and responses of individual AP+ cells (dark blue dots, *n* = 5 cells) and for AP− cells (light blue dots, *n* = 263 cells).

Next, we asked how sensory information of the air puff stimulation is transmitted to the dentate gyrus. The medial entorhinal cortex (MEC) is one of the major inputs into DG via the medial perforant path (MPP). We therefore expressed the red shifted Ca^2+^ indicator jRGECO1a in MEC excitatory cells using viral gene transfer under the CaMKII promoter in Thy1 GCaMP6 animals. This allowed us to simultaneously monitor the activity of the medial perforant path as well as granule cells, as previously described (Pofahl et al., 2021; see Materials and Methods section; Fig. 3*A*,*B*).

Air puff stimulation reliably elicited responses of the MPP in the dentate molecular layer in 86 ± 3% of all applied stimuli (Fig. 3*C*,*D*, *n* = 3 mice). In cases where the air puff did not trigger locomotion, a clear peak of MPP bulk fluorescence with a subsequent decay was observed following the stimulus (Fig. 3*C*). In cases in which mice started running after the stimulus, the initial fluorescence peak was followed by a significantly elevated level of fluorescence (Fig. 3*D*). This elevation can be explained by the correlation of the MPP input signal to running speed (Pofahl et al., 2021) which we also observed in our control data of spontaneous running onsets (Fig. 3*E*). Using ROC analysis, we found that the sharp fluorescence peak was a good classifier for the air puff stimulus in every mouse (Fig. 3*F*).

The simultaneous recording of MPP bulk fluorescence and granule cell signals allowed us to analyze the direct correlation between the MPP input and the responding AP+ granule cells as well as the other AP− granule cells. To use comparable signals for correlation analysis, we used the Δf/F signals of both MPP bulk and granule cell activity while ignoring delays >1 s which would be shaped by the respective Ca^2+^ indicator dynamics (Fig. 3*G*). Since MPP signal and AP+ cells were both correlated to the air puff stimuli, we also found a correlation between these two signals and analogously an anticorrelation between MPP and the mean signal of the AP− cells (Fig. 3*H*). Looking at the correlation of individual granule cells to the MPP input following the stimulus, we found that 4/5 of AP+ cells were strongly correlated to the MPP and 1/5 cell was weaker, but still positively correlated (Fig. 3*I*; *r* = 0.6 ± 0.26, mean ± SD). The distribution of the other granule cells was centered at *r* = −0.09 but with long symmetric tails toward positive and negative correlations (SD = 0.35). While the mean *r* value close to zero seems to contradict the clear anticorrelation of the average signal (Fig. 3*H*), this rather highlights that the inhibitory effect is difficult to grasp when looking at individual sparsely active granule cells but becomes evident when looking at the entire ensemble. Comparing the correlation coefficients of AP+ versus AP− cells, we found this difference to be significant (*n* = 5 vs 263 cells for responder and nonresponders, respectively. Subsampling with size matched groups median *p* = 0.016, 80% of *p* values < 0.05, 1,000 iterations).

Next, we asked whether the response amplitudes of granule cells would directly correlate to the variations of the MPP input amplitude following the air puff stimuli. To that end, we subtracted from each signal its mean response and calculated the correlation of the remaining amplitudes analogous to the concept of noise correlation (Averbeck et al., 2006). We did this across all stimuli between the MPP input and each granule cell. For the AP+ granule cells, we found that all noise correlation coefficients were positive but very small and not indicating a noise correlation (Fig. 3*J*; *r* = 0.08 ± 0.08, mean ± SD). For the nonactivated set of granule cells, we found a lower mean value close to zero but again a broad distribution of values (*r* = 0.01 ± 0.16, mean ± SD). Hence, in our data given the small number of AP+ cells, we did not find evidence that the response amplitude would directly correlate to the amplitude of the MPP input, which suggests that there are other factors additionally to the MPP input that influence granule cell activity.

### MPP activation mainly induces inhibition in dentate granule cells

The in vivo imaging data imply that sensory stimulation elicits strong and widespread inhibition controlling activity of the granule cell ensemble. Therefore, we next investigated the direct excitatory and inhibitory response of individual granule cells to MPP activation.

**Figure 4.**
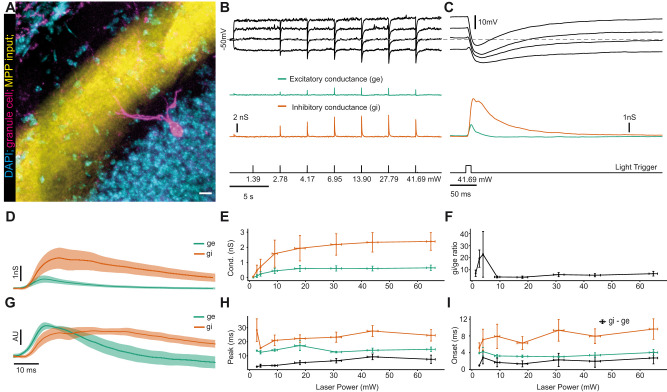
In vivo patch-clamp analysis of perforant-path-triggered excitation and inhibition in granule cells. ***A***, Post hoc reconstruction of a patched granule cell (magenta) within the hippocampal granule cell layer (DAPI stained cell bodies, cyan) and the ChR2 expressing axons from MPP (yellow). ***B***, Top panel (black), EPSP/IPSP voltage responses of the granule cell potential to light stimulation of afferent fibers recorded at four different current injections. Middle panels (green and orange), Estimated excitatory and inhibitory conductances, respectively. Bottom panel, Times and intensities (power at fiber end) of light stimulation. The dashed line depicts the resting membrane potential of −50 mV. ***C***, Close-up of the last stimulation shown in ***B***. ***D***, Average pooled responses ± SEM of excitatory and inhibitory conductance (green and orange, respectively) at maximum light stimulation intensity with SEM (*n* = 9 cells). ***E***, Pooled responses measured at different light stimulation intensities. *x*-error bars denote estimated variability in laser power at, *y*-error bars denote SEM. ***F***, Inhibition–excitation ratio calculated from ***E*** for different stimulation intensities. *x*-error bars denote estimated variability in laser power at, *y*-error bars denote SEM. ***G***, Normalized peak amplitude of conductance responses shown in ***D*** to illustrate the time course of both conductances in comparison. Excitatory conductance FWHM = 22 ± 3 ms, inhibitory conductance FWHM = 45 ± 7 ms. *x*-error bars denote estimated variability in laser power at *y*-error bars denote SEM. ***H***, Delay between the stimulation time and the peaks of excitatory and inhibitory conductance (green and orange, respectively) as well as the delay between both peaks (black). *x*-error bars denote estimated variability in laser power at, *y*-error bars denote SEM. ***I***, Delay between the stimulation time of excitatory and inhibitory to conductance response onset as well as the delay between both onsets (black). *x*-error bars denote estimated variability in laser power at, *y*-error bars denote SEM. Extended Data Figure 4-1 illustrates the goodness of fit estimation for each patched granule cell in the dataset. Extended Data Figure 4-2 illustrates the recruitment of EPSCs and IPSCs in granule cells following optogenetic MPP stimulation in acute slices. Extended Data Figure 4-3 illustrates the conductance responses and resulting E/I balance in response to different stimulation powers in granule cells in acute slices. Extended Data Figure 4-4 gives a summary of published granule cell E/I ratios in the literature.

To stimulate MPP input selectively, we expressed ChR2-mCherry under the CaMKII promoter in the medial entorhinal cortex using viral gene transfer (Extended Data Fig. 4-2*A*,*B*; see Materials and Methods). In hippocampal slices prepared from these mice, light stimulation evoked EPSCs and IPSCs. Combined application of TTX and 4-AP blocked the light-evoked IPSCs, but not EPSCs (Extended Data Fig. 4-2*C*,*D*). This demonstrates that under these conditions, MPP stimulation elicits monosynaptic EPSCs, while IPSCs are mediated polysynaptically. To study the properties of MPP-evoked responses of individual granule cells in vivo, we obtained in vivo patch-clamp whole-cell recordings from dorsal hippocampal dentate gyrus granule cells in anesthetized C57BL/6 mice that also expressed ChR2-mCherry under the CaMKII promoter in the medial entorhinal cortex (Fig. 4*A*; *n* = 9 cells, 1 cell per animal). MEC principal cell somata were optogenetically stimulated with 473 nm light via an implanted light fiber above MEC. Only cells that showed granule cell morphology through biocytin filling and sufficient ChR2-mCherry MPP expression in post hoc imaging were included in the analysis (example in Fig. 4*A*).

10.1523/ENEURO.0065-25.2025.f4-1 Figure 4-1 **Error estimation of the excitation/inhibition model** **A**, Mean squared error (MSE) of the model fit for each responding granule cell in the data set. **B**, R^2^-value of the model fit for each responding granule cell in the data set. **C,** Correlations between the input data and the reconstructed traces after the model fit for each responding granule cell in the data set. **D-L,** Example response for each patched and current clamped granule cell. Measured voltages for the four different current injections in black. Reconstructed voltage traces after the model fit in yellow. Download Figure 4-1, TIF file.

10.1523/ENEURO.0065-25.2025.f4-2 Figure 4-2 **Optogenetic MPP stimulation elicits monosynaptic EPSCs and polysynaptic IPSCs** **A**, Histology of acute coronal slice preparation of dorsal hippocampus. MPP fibers of left hippocampus express ChR2-mCherry (yellow), as evident from the fluorescent band in the middle molecular layer of the dentate gyrus. **B**, Coronal slice of MEC, showing ChR2-mCherry expressing infected cells in superficial layers. **C,** Light-evoked PSCs at different membrane potentials, allowing discrimination of IPSCs (at 0 mv) and EPSCs (at -80 mV, upper trace). Application of TTX (1 µM) and 4-AP (200 µM) to isolate monosynaptic PSCs abolished IPSCs, but not EPSCs. **D,** Gabazine application (10 µM) abolishes the IPSC, but not the EPSC (n = 5). **E,** Quantification of the effects of combined application of TTX and 4-AP (n = 5) on EPSC and IPSC peak amplitudes. **F,** Dependence of inhibitory and excitatory conductances on the laser power. Recordings in this figure were performed at a laser power eliciting maximal PSC amplitudes. Error bars denote SEM. Download Figure 4-2, TIF file.

10.1523/ENEURO.0065-25.2025.f4-3 Figure 4-3 **Properties of MPP evoked inhibition and excitation in the slice preparation** **A**, Representative excitatory conductance (green) and inhibitory conductance (orange) in response to light stimulation. Inhibitory conductances are smaller and have slower kinetics compared to excitatory conductances. Lower traces normalized to the same peak conductance to illustrate the difference in kinetics. Shaded areas denote SEM. **B**, Peak excitatory conductance (green) and inhibitory conductance (orange) in response to different light stimulation intensities. Error bars denote SEM. **C**, Inhibition to excitation ratio for different light stimulation intensities. Error bars denote SEM. **D**, Time to peak of excitatory conductance (green) and inhibitory conductance (orange) in response to different light stimulation intensities. Error bars denote SEM. **E,** Time from stimulation to response onset of excitatory conductance (green) and inhibitory conductance (orange) in response different light stimulation intensities. Error bars denote SEM. Download Figure 4-3, TIF file.

10.1523/ENEURO.0065-25.2025.f4-4 Figure 4-4 **Granule cell E-I ratios in the literature**, E-I ratios as found in different publications (Citation), the method used, the technique used to isolate inhibitory and excitatory currents/potentials/conductances, the estimated inhibition-excitation ratio and the frequency dependence of the ratio. Download Figure 4-4, DOCX file.

Optogenetic MPP stimulation elicited a brief low-amplitude depolarization followed by a hyperpolarization of larger amplitude (Fig. 4*B*). This was true at different membrane potentials when adjusted with constant current injection. Of note, larger amplitude hyperpolarizations were also observed at membrane potentials comparable to those reported previously (Pernía-Andrade and Jonas, 2014).

To isolate excitatory (*g_e_*) and inhibitory conductances (*g_i_*), we altered the membrane potential of granule cells to four different levels using constant current injections. At each of these potentials, we stimulated perforant path-evoked synaptic potentials with 10 ms pulses of blue light (Fig. 4*B*). At different current injection magnitudes, synaptic response voltage waveforms are altered because of the difference in membrane potential (Fig. 4*C*). Using these different waveforms, *g_e_* and *g_i_* can be estimated throughout the time course of the postsynaptic potentials (Priebe and Ferster, 2005; see also Materials and Methods). The goodness-of-fit for this model was estimated for every cell which confirmed a good correspondence between data and model result (Extended Data Fig. 4-1). We found that the inhibitory conductance was much larger than the excitatory conductance for all cells (Fig. 4*D*, data shown for maximal laser power). This was true over a range of stimulation intensities (Fig. 4*E*,*F*, asterisks indicate one-sample *t* tests against 1, *g_i_*/*g_e_* ratio at maximum stimulation power 6.7 ± 2.1).

Aside from the peak magnitude of conductance changes, the relative time course of *g_e_* and *g_i_* strongly determines the likelihood and timing of action potential generation in granule cells. The peak normalized averaged waveforms at the highest laser power showed a short onset delay of roughly 2 ms (Fig. 4*G*). Quantifying the time lag between *g_e_* and *g_i_* peak revealed a lag of 7.5 ± 3.2 ms (Fig. 4*H*) at the highest laser power and an onset delay of 2.7 ± 1.5 ms (Fig. 4*I*). These short time delays are consistent with the expected strong contribution of feedforward inhibition. Comparing the time courses of the inhibitory and the excitatory conductances, we found that *g_e_* had a FWHM_e_ = 22 ± 3 ms, which was much shorter than the one of *g_i_* with FWHM_i _= 45 ± 7 ms. This suggested a longer lasting inhibitory response compared with the excitatory response.

Taken together, we conclude that MEC stimulation evokes strong, long-lasting inhibition with a short, millisecond scale delay after excitation onset.

### Excitation–inhibition balance across different stimulation frequencies

**Figure 5.**
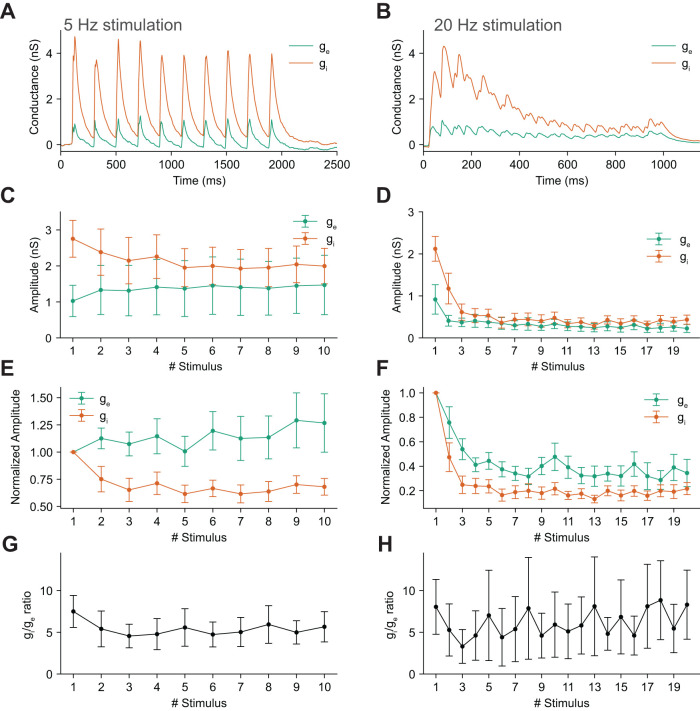
Excitation–inhibition balance in granule cells during repetitive stimulation of perforant path in vivo. ***A***, ***B***, Representative excitatory and inhibitory conductance traces during 5 and 20 Hz stimulation, respectively. ***C***, At 5 Hz excitatory conductance is slightly facilitating while inhibitory conductance is depressing. Error bars denote SEM. Friedman test: *n* = 9, df = 9, *g_e_*: *χ*^2^ = 5, *p* = 0.87; *g_i_*: *χ*^2^ = 22, *p* < 0.01. ***D***, At 20 Hz both excitatory and inhibitory conductance are strongly depressing. Error bars denote SEM. Friedman test: *n* = 7, df = 19, *g_e_*: *χ*^2^ = 55, *p* < 0.0001; *g_i_*: *χ*^2^ = 40, *p* < 0.0001. ***E***, Like ***C*** with amplitudes normalized to first elicited conductance. Error bars denote SEM. *n* = 9 cells. ***F***, Like ***D*** with amplitudes normalized to first elicited conductance. Error bars denote SEM. *n* = 7 cells. ***G***, At 5 Hz inhibitory peak conductance is consistently larger than excitatory during the entire stimulus train. Error bars denote SEM. Friedman test did not show a significant effect of stimulus: *χ*^2^ = 8.03, *n* = 9, df = 9, *p* = 0.53. ***H***, At 20 Hz the inhibitory peak conductance is also consistently larger than the excitatory during the stimulus train. Error bars denote SEM. Friedman test did not show a significant main effect for stimulus: *χ*^2^ = 25.01, *n* = 7, df = 19; *p* = 0.16. Extended Data Figure 5-1 illustrates the E/I balance in responses to frequency stimulation in granule cells in acute slices.

To test the frequency dependence of the excitation–inhibition (I/E) balance, we studied repetitive stimulation protocols. Theta-band stimulation (5 Hz) responses led to stable excitatory responses over 10 stimulations (Friedman test, *χ*^2^ = 4.61, *p* = 0.87), while inhibitory responses decreased (Friedman test, *χ*^2^ = 21.75, *p* = 0.0097, Fig. 5*A*,*C*,*E*). Faster stimulation (20 Hz) led to a depression in both excitation and inhibition (Friedman test, excitation: *χ*^2^ = 55.45, *p* = 1.98 × 10^−5^, inhibition: *χ*^2^ = 34.89, *p* = 0.014, respectively; Fig. 5*B*,*D*,*F*). When we computed the ratio of *g_i_* to *g_e_*, however, the different dynamics of the two conductances were not sufficient to cause significant differences (Fig. 5*G*,*H*; Friedman test, 5 Hz: *χ*^2^ = 8.03, *p* = 0.53, 20 Hz: *χ*^2^ = 25.01, *p* = 0.16). We conclude that there are reductions in *g_i_* during repetitive stimulation that may lead to subtle changes in I/E balance. The latter finding was not statistically significant; however, we note that the statistical power of our analysis was low (0.082 for 5 Hz and 0.33 for 20 Hz).

10.1523/ENEURO.0065-25.2025.f5-1 Figure 5-1 **Excitation-inhibition balance in granule cells during repetitive stimulation of perforant path in the slice preparation.** **A,** Time courses of excitatory conductance (green) and inhibitory conductance (orange) for 5 Hz, 10 Hz, 20 Hz and 30 Hz stimulation trains. **B**, Mean conductance amplitudes across granule cells. Error bars denote SEM. Friedman tests for the changes of amplitudes: 5 Hz: n = 11, df = 9, ge: χ^2^ = 54, p < 0.0001; gi: χ^2^ = 45, p < 0.0001; 10 Hz: n = 12, df = 9, ge: χ^2^ = 67, p < 0.0001; gi: χ^2^ = 40, p < 0.0001; 20 Hz: n = 12, df = 9, ge: χ^2^ = 61, p < 0.0001 ; gi: χ^2^ = 38, p < 0.0001; 30 Hz: n = 11, df = 9, ge: χ^2^ = 35, p < 0.0001; gi: χ^2^ = 52, p < 0.0001. **C**, Like B with amplitudes normalized to the peak amplitude of the first PSC in the train. Error bars denote SEM. **D**, Inhibition to excitation ratio for different stimulation frequencies. Error bars denote SEM. Friedman test, 5 Hz: χ^2^ = 15, n = 11, df = 9, p = 0.09; 10 Hz: χ^2^ = 11, n = 12, df = 9, p = 0.25; 20 Hz: χ^2^ = 37, n = 12, df = 9, p < 0.0001; 30 Hz: χ^2^ = 31, n = 11, df = 9, p < 0.001. Download Figure 5-1, TIF file.

### Comparison to in vitro analysis of MPP-evoked inhibition

In parallel to the in vivo patch-clamp recordings, we also examined recruitment of granule cells by selective optogenetic MPP activation in the slice preparation (Extended Data Fig. 4-2*A*,*B*). Inhibitory and excitatory currents were measured in voltage-clamp mode and pharmacologically isolated (Extended Data Fig. 4-2*C*,*D*,*E*; see Materials and Methods). Of note, while stimulation of MPP fibers was carried out optogenetically both in vivo and in vitro, the pharmacological isolation could only be carried out in the in vitro condition.

For optogenetic stimulation in the slice, the light stimulation was focused on the terminal region in the medial molecular layer of the dentate gyrus. The optogenetically evoked responses saturated at light powers comparable to the in vivo experiments (Extended Data Fig. 4-2*F*). The respective inhibitory and excitatory conductances were calculated using the reversal potentials (Extended Data Fig. 4-3*A*,*B*). The kinetics were generally comparable to the in vivo experiments, with similar onset times of the inhibitory and excitatory conductances (Extended Data Fig. 4-3*C*; excitatory conductance onset time: *F*_(1,16)_ = −1.91, *p* = 0.07, inhibitory conductance onset time: *F*_(1,19)_ = −1.55, *p* = 0.14). The same was true for the time to peak of the inhibitory conductance, while the rise for the excitatory conductance was faster in vitro (Extended Data Fig. 4-3*D*; excitatory conductance time to peak: *F*_(1,16)_ = −6.39, *p* < 0.01, inhibitory conductance onset time: *F*_(1,19)_ = −1.71, *p* = 0.10). A difference was apparent when computing the I/E ratio, which was more than an order of magnitude lower than that found in our in vivo granule cell recordings (Extended Data Fig. 4-3*E*, Fig. 4*F*; <0.5 in vitro vs 7.50 to 5.66 in vivo, *F*_(1,19)_ = −3.2, *p* < 0.1).

We further examined the dynamics of inhibitory and excitatory responses, as well as I/E ratios during repetitive stimulation in vitro. In the in vitro condition, both *g_i_* and *g_e_* were reduced significantly during stimulation trains of any frequency (5–50 Hz; Extended Data Fig. 5-1*A–C*; Friedman test, statistics for all frequencies see figure legend Extended Data Fig. 5-1). Comparable to in vivo recordings, the I/E balance was maintained in in vitro recordings during stimulation trains at both 5 and 10 Hz (Friedman test, n.s.; Extended Data Fig. 5-1*D*). At higher stimulation frequencies, however, the I/E ratio was significantly increased (Friedman test 20 Hz: *χ*^2^ = 36.98, *p* = 0.00003; 30 Hz: *χ*^2^ = 31.48, *p* = 0.0002; Extended Data Fig. 5-1*D*; statistics for all frequencies see figure legend Extended Data Fig. 5-1).

## Discussion

We show that mild aversive air puff stimulation during immobility triggers an activity cascade through the medial perforant path to the dentate gyrus. While this stimulation leads to a significant direct excitation of a small subset of granule cells (2.6% of recorded cells), the effect on the other granule cells is a 1-s-long lasting inhibition of activity. Further, we show that direct optogenetical stimulation of medial entorhinal cortex cells leads to a fourfold stronger inhibition than excitation in individually patched granule cells in anesthetized mice. This strongly biased E/I balance is also stable for stimulation different frequencies in vivo. Taken together, our findings shed light on the inhibitory effects acting on dentate gyrus granule cells which are necessary to maintain sparse activity levels which are thought to be the basis of pattern separation (Cayco-Gajic and Silver, 2019).

Across brain regions and species, inhibitory circuits shape neuronal population activity. In the hippocampal dentate gyrus, inhibition promotes sparse granule cell activity, which in turn contributes to the function of pattern separation. Numerous studies have examined the circuit properties of inhibition in the dentate gyrus in hippocampal slices, leading to a refined model of the circuit basis of inhibition (Strüber et al., 2015, 2017; Espinoza et al., 2018; Braganza et al., 2020; Guzman et al., 2021).

However, there is substantial intrahippocampal connectivity within the dentate gyrus that exceeds the dimensions of a typical slice preparation (Buckmaster and Schwartzkroin, 1995; Sik et al., 1997; Bienkowski et al., 2018; Yen et al., 2022). Thus, the properties of inhibition recruited in vivo may be different from those predicted by local inhibitory circuits. For this reason, assessing inhibition of the dentate gyrus in in vivo experiments is important and relevant. We have used both in vivo imaging and in vivo patch-clamp recordings to assess the properties of inhibition in the dentate gyrus on the population and single-neuron level. Our data show that medial perforant path stimulation selectively activates a sparse set of granule cells while simultaneously causing widespread inhibition across the remainder of the granule cell population. In addition, we show with in vivo whole-cell recordings that medial perforant path-triggered inhibition is large, fast, and maintained during repetitive stimulation.

Our two-photon in vivo imaging data show that on average only 2.6% of dentate gyrus granule cells respond significantly to sensory-evoked MPP activation. Because even this responding subclass of granule cells responds unreliably to air puff stimulation, this means that the fraction of responding cells activated after a given air puff is very low. Nevertheless, the remaining granule cells were on average suppressed over a time window of up to one second. This is generally consistent with the sparse firing of granule cells observed with both imaging and in vivo electrophysiology (Danielson et al., 2016; Diamantaki et al., 2016; Pilz et al., 2016; GoodSmith et al., 2017; Senzai and Buzsáki, 2017; Hainmueller and Bartos, 2018; van Dijk and Fenton, 2018). A similar phenomenon has also been observed for cue-responsive DG populations, which—when activated—also lead to significant inhibition of spontaneous and place-related firing of the remaining granule cell population (Tuncdemir et al., 2022). The strong contrast in granule cell responses with sparse activation and widespread inhibition is in line with a “k-winners take it all” model that has been proposed for the DG network (O'Reilly and McClelland, 1994; de Almeida et al., 2009; Braganza et al., 2020).

Our design aimed at investigating sensory-related responses without additional behavioral confounds. We therefore limited our stimulation time points to resting periods, to exclude space and speed as confounding factors. We also randomized stimulation in time and space to prevent temporal or spatial learning. Still, we observed differences in granule responses depending on whether locomotion was initiated by the air puff stimulation or not. The response amplitudes responses were no significantly different in either scenario or for the AP+ or the AP− cells. The dynamics of the response without running initiation were fast with a FWHM 262 ± 30 ms for the AP+ cells, while they were much slower for the same cell population with running onsets. We cannot conclusively dissect how much of the late excitation is caused by the air puff versus running. However, it is likely that the late excitation is due to locomotion, because similar prolonged excitation was observed in pure running onsets without air puff stimulation both in the granule cells and the MPP bulk signal.

The simultaneous recordings of air puff responses in MPP bulk input and individual granule cell activity illustrate the general correlation in this feed forward network. One feature hypothesized for the dentate gyrus is the orthogonalization of its inputs which underlies the concept of pattern separation (Cayco-Gajic and Silver, 2019). While our data showed the direct correlation of MPP input to the overall response in the granule cells, we did not observe a correlation of response amplitude to the variations of the input amplitude. This suggests that for the AP+ cells that we measured, the individual stimulus-wise response amplitude could also be influenced by other inputs or modulatory factors. Given the small number of AP+ cells in our paired recordings, this conclusion must be interpreted with caution, and we cannot exclude that correlations might become apparent with more cells. However, the lack of correlation is in line with the general observed unreliability of granule cell responses with no granule cell responding to more than two-thirds of stimuli and 18% of all granule cells responding exactly once. Taken together, these data could shed light on how the dentate granule cell network generates unique output patterns from one of its input sources.

While the in vivo imaging experiments serve only as an indirect measure of the inhibitory effects onto granule cells, our whole-cell recordings of granule cells directly show the strong inhibitory conductance change triggered by MPP activation. There were several remarkable features of DG inhibition. Firstly, inhibition was large, with high inhibition–excitation ratios. This is consistent with prior work describing prominent IPSPs in granule cells following MPP activation (Buckmaster and Schwartzkroin, 1995), although this study did not determine inhibition–excitation ratios quantitatively. In vitro, on the other hand, we found excitation to be larger than inhibition. We note that this is in contrast to previous slice work. Ewell and Jones (2010) found IPSCs to be approximately three times larger than EPSCs and Marín-Burgin et al. (2012) find them to be approximately twice as large (see summary of published work in Extended Data Fig. 4-4). We surmise that this could be due to methodological differences. Firstly, we have isolated excitatory and inhibitory PSCs with gabazine in every individual experiment, while previous studies relied on voltage clamping to the respective reversal potentials. Secondly, we stimulated MPP using optogenetic approaches in both our in vivo and in vitro recordings, while previous in vitro studies employed electrical stimulation. Although some studies used AMPAR blockers (Ewell and Jones, 2010) to exclude direct stimulation of GABAergic axons, this was not done in all studies and experiments. Direct stimulation of GABAergic axons would be expected to enhance the measured inhibition. Other factors also affect I/E balance, for instance, if recordings were done from dorsal or ventral hippocampus or recording temperature. The latter is directly addressed in one paper (Hsu et al., 2016), which clearly shows much smaller I/E ratios at body temperature similar to the in vivo condition (0.4 at 34° vs ∼2 at 23°C; see Extended Data Fig. 4-4). However, even given the diverse values of I/E balance reported under different conditions in vitro, the I/E ratio we measured in vivo is still roughly twice as large as the largest ratio measured in vitro.

We also examined the dynamic features of inhibition–excitation ratios. We found that in vitro, stimulation frequencies ranging from 5 to 50 Hz caused frequency-dependent decreases of both inhibition and excitation. At frequencies >10 Hz, excitatory responses showed larger decreases than inhibition, leading to dynamic increases in I/E ratios. These in vitro findings are in line with published work (Marín-Burgin et al. (2012), their Fig. S8; Pardi et al. (2015), their Fig. 3 Supplement 2; but see Hsu et al., 2016]. In our in vivo experiments, inhibitory responses were significantly depressed at 5 and 20 Hz, whereas excitatory responses showed significant depression only at 20 Hz. Despite these differential changes, I/E ratios were not significantly altered during repetitive stimulation at any frequency. We note that the statistical power of these analyses of I/E ratios was low, and larger sample sizes could reveal reductions of I/E balance during repetitive stimulation. Nonetheless, the fact that in vivo I/E balance is either unchanged or potentially reduced under some conditions appears different from our and previous in vitro studies (Marín-Burgin et al., 2012; Pardi et al., 2015) showing increased I/E ratios during stimulation trains.

Decreased I/E ratios due to stronger adaptation of inhibition compared with excitation have been observed in sensory cortex and has been proposed to act as a gain mechanism active during sustained sensory activity (Heiss et al., 2008; Cohen-Kashi Malina et al., 2013). In the dentate gyrus, previous studies have proposed that this region has frequency-dependent filtering properties (Hsu, 2007; Ewell and Jones, 2010; Marín-Burgin et al., 2012; Scullin and Partridge, 2012; Pardi et al., 2015; Lee et al., 2016). Specifically, measurements of pre- and postsynaptic activity suggest that the dentate gyrus may act as a low-pass filter (Scullin and Partridge, 2012), consistent with our observation of reduced inhibition but not excitation during low-frequency 5 Hz activation. At higher (20 Hz) stimulation frequencies, both inhibition and excitation are reduced. This frequency range is relevant to cognition, as novelty experience and certain types of exploratory activity are associated with increased activity in the gamma or beta frequency bands (Rangel et al., 2016; Trimper et al., 2017; Barth et al., 2018). Moreover, gamma frequency-related communication between the entorhinal cortex and dentate gyrus support specific types of memory synchronization (Fernández-Ruiz et al., 2021). Finally, pattern separation has been proposed to operate particularly efficiently at gamma frequencies, due to frequency-dependent properties of inhibitory feedback circuits (Braganza et al., 2020).

Finally, we show that inhibition in DG is fast, lagging excitation by only 5–10 ms on average. While we cannot dissect contributions of feedforward and feedback inhibition rigorously, it is likely that there is a strong contribution of feedforward inhibition to initial phases of MPP-induced inhibition. Indeed, activation of some types of interneurons in the dentate gyrus can even precede the activation of granule neurons (Li et al., 2013).

We note that the inhibition assessed by direct patch-clamp recordings was in the order of hundreds of milliseconds, while the inhibition of neuronal activity seen in in vivo imaging experiments lasts up to a second. Slow IPSPs in granule cells have been described (de Koninck and Mody, 1997; Mircheva et al., 2019). Because our in vivo recordings required the intracellular presence of QX314, which blocks GABA_B_-activated K^+^ channels (Nathan et al., 1990; Andrade, 1991), it is possible that our patch-clamp recordings underestimate the duration of synaptically evoked inhibition after MPP activation. Further, the lasting activation of some granule cells that we have seen in our data could also lead to a further recruitment of feedback inhibition, which could lead to the long-lasting inhibition of the nonresponding granule cells.

In summary, we show that MPP input evokes fast and strong inhibition that causes widespread depression of granule cell population activity. This may be relevant for pattern separation, a key proposed function of the dentate gyrus.

## Synthesis

Reviewing Editor: Mark Sheffield, University of Chicago Division of the Biological Sciences Decisions are customarily a result of the Reviewing Editor and the peer reviewers coming together and discussing their recommendations until a consensus is reached. When revisions are invited, a fact-based synthesis statement explaining their decision and outlining what is needed to prepare a revision will be listed below. The following reviewer(s) agreed to reveal their identity: Antoine Madar, Thibault Cholvin.

The manuscript presents a comprehensive study of medial perforant path (MPP) inputs to dentate granule cells (GCs), integrating in vivo two-photon calcium imaging with in vitro and in vivo patch-clamp recordings. Both reviewers recognize the significance of the study in elucidating the inhibitory-excitatory balance in the dentate gyrus and its relevance to circuit-level computations such as pattern separation. However, several methodological and analytical concerns must be addressed to strengthen the manuscript's conclusions.

A primary concern is the identification and classification of airpuff-responsive GCs. The current criteria for defining responsive cells require further justification, and alternative analytical approaches, such as generalized linear models (GLMs) or decoding analyses, may provide a more principled framework. Additionally, the impact of behavioral and internal state variables, such as pupil size and locomotion, on DG activity is underexplored. A deeper analysis of these factors could enhance the understanding of DG responses during behavior.

Another major issue is the interpretation of the inhibitory/excitatory (I/E) ratio dynamics. The claim that the I/E ratio remains stable across frequencies contradicts prior studies, and the estimation method should be further validated. Reviewers also note potential concerns about the accuracy of the model used to infer conductances, emphasizing the need for additional data presentation, including error estimates and model fit evaluations.

Several methodological clarifications are also needed, including details on electrode placement, airpuff presentation, and histological verification of viral expression. Additionally, the study should address potential consequences of CA1 aspiration on DG circuit function. Improvements in data visualization, statistical approaches, and figure organization would further enhance the manuscript's clarity and rigor.

Both reviewers agree that the study presents valuable findings but requires substantial revisions, particularly in refining the analytical framework and improving methodological transparency.

## Author Response

Rebuttal letter We sincerely thank both reviewers for their thoughtful and constructive feedback. Their insights helped us identify important blind spots and prompted us to critically reassess several aspects of our approach and analysis, which were central to addressing our research question. In response, we have substantially revised the first three sub-sections of the Results section, resulting in a clearer and more coherent narrative. Additionally, we incorporated one new main figure, two new supplementary figures, and updated figure panels across all original figures. To further improve transparency and reproducibility, we expanded the Methods section with additional details. Overall, we believe these revisions have significantly strengthened the manuscript by providing clearer justification for each analysis step and a more logically motivated progression of the study.

Reviewer 1 For clarity I refer to line numbers, which I counted from the first line of the introduction, without counting line spaces between paragraphs, but including figure legends. In the future, please make it easier on the reviewers and number the lines of your manuscript.

We have taken this to heart and have added line numbers to the revision.

Summary: This study comprises 3 different experiments in mice: 1) simultaneous 2-photon calcium imaging of dentate granule cells (GCs) and bulk medial perforant path (MPP) inputs in behaving mice receiving occasional airpuffs when they stopped running; 2) in-vitro and 3) in-vivo patch-clamping of single GCs to evaluate the inhibition/excitation ratio during repetitive optogenetic stimulations of the MPP. The authors confirm the long-standing view that EC projections drive DG neurons, including a strong inhibition to maintain sparse activity in GCs. They also make surprising claims that the dynamics of the I/E ratio are stable and independent of input frequency, contrasting with past slice studies, which would be very interesting if it was better supported by the data. The first experiment offers a rich dataset that could be analyzed more deeply to better understand what the dentate gyrus encodes.

Main comments 1) First paragraph in result section: The analysis of behavioral variables is insufficient and seems out of place (the goal of recording speed or pupil size is never spelled out, and the pupil size data is not interpreted). It seems better related to the next section arguing that "Granule cells response are not correlated to running initiation".

The in-vivo dataset is rich, with simultaneous recordings of bulk MPP signal, single GCs and behavioral variables like speed, acceleration (position too?) and pupil size telling us about internal arousal states. The current story is about sparsity and inhibition of DG responses, mostly confirming established ideas, but many interesting questions somewhat outside of this framework could be asked to more fully characterize DG responses during behavior. Regarding the response to airpuffs, at the center of the study, why do some GCs respond sometimes and not others? Can the probability of response be linked to any behavioral or neuronal variables? Are there any correlations between cells of the same animal in the emergence trial of the response? and the termination trial (i.e. is it cell-specific or animal specific)? More generally, what is DG encoding? Is the DG response dependent on locomotive or internal states? Is there mixed selectivity or clearly separate populations distinctly coding for airpuffs, speed/acceleration, pupil size? Fig S1 is an appreciated first pass regarding the impact of locomotion, but it is based on average responses and the assumption that there are 2 distinct populations of responsive and non-responsive cells (see comment #7). Similar plots distinguishing between trials with pupil response and no response could be produced to complement Fig S1 as a first pass on the question on the role of internal states on DG activity. But additional approaches relying on less or different assumptions are needed as well. For instance, a linear mixed-effects model based on the full dataset (e.g. DG signal ~ Airpuff*Speed*Pupil + (1+ Speed|mouse)) would allow to test for interactions between different predictor variables and account for individual variability (cell id could also be nested). A Poisson GLM encoding model would allow to describe cell-wise responses as a function of all kinds of predictor variables (as in Engelhard and Witten 2019 for example. See also Aljadeff and Kleinfeld 2016). Alternatively, a multidimensional approach (e.g. quantifying the receptive field of GCs in Speed x Pupil x Sensory space) akin to what Nieh and Tank (2021) used (see their Fig 2 and Fig S2) could address similar questions with less assumptions.

We thank the reviewer for the very careful perusal of our manuscript and the thoughtful and constructive suggestions. As the questions are somewhat interrelated, we have tried to answer them comprehensively below.

Indeed, we did not point out the reasons for recording behavioral parameters sufficiently. We corrected this in the current version of the manuscript. Regarding the pupil sizes, we used these to test if air puffs are effective sensory stimuli. The air puffs were weak, and directed at the animals' backs, and thus does not constitute a particularly aversive or strong stimulus. The pupil constriction was the readout we used to verify that the animal reacts to the air puff and is therefore crucial at the beginning of the results section. We have made this part more explicit and have also clarified that only air puff stimuli that were followed by a significant pupil constriction were used in further analysis. We added panels showing the ensemble signals and behavioral readouts for those stimuli that did not trigger significant pupil constriction to the supplement as suggested by the reviewer (Fig. 1-1F-I). Also, we added a ROC analysis to show that the pupil constriction is a good classifier for the air puff (Fig. 1E). We did not conduct further comparisons between trials with and without pupil constriction, as we reasoned that this could constitute circular argumentation.

In this paper, our intent in using air puff stimulation was to trigger a physiological input signal into the dentate gyrus. At the same time, we wanted to minimizing or pre-define other confounding behavioral factors. This is why we chose to stimulate only during resting states, since hippocampal activity differs between resting and locomotion and we and others have described for the dentate (Danielson et al., 2016; Pilz et al., 2016; Pofahl et al., 2021). Further, we randomized the timing of air puffs and applied them wherever the animal rested on the belt to prevent temporal anticipation or spatial learning. Hence, in this study we did not center the analysis on the impact of multiple behavioral factors on the airpuff response. Nevertheless, we acknowledge that we cannot eliminate all behavioral factors and therefore followed the reviewer's suggestion to apply linear mixed-effects models by using the standard MATLAB procedures and the code supplied by (Engelhard et al., 2019). We used absolute pupil size, change of pupil size and running initiation as inputs. However, in our hands these procedures did not yield clear results towards the tuning of individual cells or mice with one of these variables. This can have many reasons, such as the sparsity of granule cell activity when we use the event onsets, the noise of the data when we use Df/F traces as inputs, the individual differences between mice regarding whether running was triggered or not. Since our results are ambiguous, we do not think that reporting these approaches would add clarity to our study.

However, since there are individual differences between mice and the dentate activity indeed changes when an animal initiates locomotion, we added figure panels to highlight mouse-wise analyses (Fig. 1E, Fig. 1-1 A, B, E, K, L, N, O, P, Fig. 2-1 A, B, D), and we emphasized the differences between trials with and without running initiation by making the former Suppl. Fig. 1 a main figure and by adding further analysis (Fig.2 E-T, Fig.2-1 A-E).

2) A lot of the argument of the paper is based on the identification of signals triggered by airpuffs. I find the methodology to define airpuff-responsive cells/populations (GCs or MPP) problematic in several regards. First, the shuffling procedures used for controls or for defining significance need to be more detailed and clarified (both in results/figure and methods). It is currently not clear what was shuffled and how. The thresholds used to define significance (e.g. 4 responsive laps for GC puff cells) also seem arbitrary and need justifications. Does using different thresholds for puff-responsive GCs change conclusions (e.g. in Fig 1i, how do distributions look like for more or less stringent criteria?)? The goal being to define airpuff-selective cells (akin to defining place cells, in a way), I wish the approach was more principled, for instance based on the distributions of true and false positives (% of puff trials with/without a response) and negatives (% of immobility periods without/with a response), using a ROC analysis. As far as I understand, the current method to define airpuff-responsive GCs does not consider GC responses during periods where the animal paused without an air puff. An alternative would be to use a decoding or information-theoretic approach to assess the "puff-information score" of individual cells, based on the full activity trace of each cell (not just a narrow time window around puffs) and then use a score threshold (informed by the distribution of scores) to define puff-responsive cells.

We thank the reviewer for pointing this out. We have added further explanation on how the shuffled distributions are produced in the methods section. The criterion so far was based on the response probability exceeding the 95th percentile of the shuffled distribution for every individual cell. The hardcoded threshold of three responded events had the purpose of filtering GCs with low response probability which could anyway be significant given the overall sparse activity of GCs. We acknowledge that a hardcoded threshold is not elegant especially when the number of administered air puffs differs between animals. Hence, this threshold has been replaced with a dynamic threshold of 5% of air puffs that a given GC had to exceed. This change in definition did not change the identity of identified responders.

However, we appreciate that the suggested alternative responder definitions could be more principled. Therefore, we implemented all the definitions that were suggested by the reviewer into the analysis code base, which will be published with the study. The code allows now to choose an individual or combination of responder definitions to produce the main results and figure panels. The responder definitions are namely percentile of shuffled distribution with absolute or dynamic thresholding, GLM analysis, self-informed information score, and ROC analysis. All methods identify overlapping groups of responding cells with slight differences for each method (Rev. Fig. 1A). The main difference is due to the way each method handles low activity GCs. When the dynamic threshold is added to each method the amount of agreement between the methods increases (Rev. Fig. 1B). Interestingly, the main results do not change qualitatively but only quantitatively for the different definitions. To illustrate that we produced the main panels from figures 1-3 using the different responder criteria (Rev. Fig. 1C-J). This could be a hint that the strong responders that were convergently identified by all methods are also the most crucial ones. For the study, we settled on one of the methods for which we chose the ROC analysis. The ROC analysis is not sensitive for low activity cells and does not require an additional threshold. Also, it seems to be the most conservative and the most intuitive responder definition. Therefore, we switched to ROC analysis to define individual GC responder and further also the response properties of the GC ensemble, the pupil response, and the MPP response the air puff. For clarity in wording, we defined the group of significantly responding granule cells AP+ cells and all other granule cells AP- cells. We don't think that these are distinct populations of granule cells and defined the AP+ cells solely based on their responsiveness, which we also pointed out in the manuscript.

To be honest, I'm not even sure that rigorously defining airpuff cells, which necessarily entails a threshold, is critical to drive the point that GCs responses to sensory information are sparse: an analysis not relying on significance thresholds and just describing the distributions of responsive trials per cells (e.g. proba of responding), the distribution of average response amplitude, etc... may be enough. This would be especially appropriate if there is more a spectrum of more or less mixed responses than 2 clusters of responsive vs non-responsive cells (something the study currently assumes but does not demonstrate). It would indeed be interesting to describe the degree of mixed selectivity in GCs (see comment #1).

We followed the reviewer's suggestion to perform our analysis on the GC ensemble without the division into responder and non-responder. We added a pie chart showing how many granule cells never responded, non-significantly responded and significantly responded (Fig. 1L). This panel demonstrates that 68% of all imaged granule cells never respond. For all granule cells that respond at least once to an air puff we added panel showing the probability of responding (Fig. 1I) illustrating that most cells respond to very few air puffs. Further we added a panel showing the number of active granule cells after airpuff stimulation (Fig. 1H). This panel shows that - after an air puff - a lower fraction of granule cells is active compared to other resting periods. This motivated us to add another panel showing the activity time course of all granule cells around the airpuff stimulations (Fig. 1N). This panel shows that the activity does not seem to change after stimulation when looking at the entire population. We therefore added a ROC analysis using the average activity of all granule cells in each mouse (Fig. 1Q &Fig. 1-1N). This analysis showed that it is not possible to decode the air puff time form the averaged activity. When performing a ROC analysis on the average of responding AP+ cells or the AP- cells we find that both can serve as a classifier for the air puff stimulation (Fig. 1O,P &Fig. 1-1O,P). The value for the AP- granule cells is quite close to .5 but the difference is still significant. Taken together, the added analysis show that dentate gyrus seems to maintain a stable activity level when stimulated. This further illustrates that the focus on responding granule cells is indeed important to illustrate what is going on in the granule cell network. To decrease the impression that the non-responding cells form some sort of independent ensemble, we explicitly stated that our separation in significantly responding granule cells and other granule cells is solely based on the response to a single type of stimulus.

Line 158 (85% of trials with responsive MPP signal): What about the specificity of the activation to airpuffs (i.e. excluding responses due to running or else)? Again, a more principled approach like GLM or ROC analysis would make interpretations easier and potentially more interesting.

We added a ROC analysis for the MPP signal which revealed that the amplitude increase after the air puff stimulation can serve as a classifier for the airpuff (Fig. 3F). We also added figure panels that show the difference of MPP input for the different scenarios of locomotion initiation (airpuff without locomotion initiation, airpuff with locomotion initiation, locomotion initiation without airpuff, Fig. 3C-E). The correlation of MPP input to locomotion per se has been discussed on our former study using the same mice (cite Pofahl 2021). The MPP bulk signal is correlated to the running speed. However, our data also show that in the presence of an airpuff, there is an additional signal peak that is clearly faster and sharper, and thus well-separable from the signal increases caused by running initiation.

Rev. Fig. 1: Different definitions for responding granule cells A, Venn diagram depicting the quantity of identified responding granule cells and the overlap between different identification methods. Circles correspond to a shuffle criterion (blue), a glm based method (yellow), an info-score based method (orange), and a ROC based method (purple).

B, Same as A but with an additional threshold of 5% of responded stimuli. Note, the circle for the ROC based method does not change with the additional threshold.

C, Fractions of responding granule cells with different identification methods corresponding to Fig.1L. The methods from left to right are Shuffle criterion, Shuffle with threshold criterion, glm method, info-score method and ROC analysis. The methods differ in the amount of responding granule cells that are identified as significant.

D, Ensemble response of AP+ granule cells for each method (analogous to panel C) corresponding to Fig. 1O lower panel. For each method, a clear peak is visible that differs in amplitude.

E, Ensemble response of AP- granule cells for each method (analogous to panel C) corresponding to Fig. 1P lower panel. For each method, a clear peak is visible that differs in amplitude.

F, ROC analysis curves for the ensemble signals of granule cells for each method (analogous to panel C) corresponding to Fig. 1Q. The general trend is similar for every used method.

G, Mean time courses for individual AP+ granule cells for each method (analogous to panel C) corresponding to Fig.2B.

H, Ensemble Df/F signals for granule cell ensemble signals and MPP bulk input corresponding to Fig. 3G. AP+ cells identified with different methods (analogous to panel C). The general trend is visible for every used definition.

I, Cross correlation of the DF/F traces for each methods (analogous to panel C) corresponding to Fig. 3H.

J, Correlation coefficients between MPP response amplitudes and responses of individual AP+ cells for different methods (analogous to panel C) corresponding to Fig. 3I.

3) Line 56 "we found in every animal a sparse subset of granule cells that respond to air puff stimulation (Fig. 1G), with in total 52/1583 granule cells classified as responders (corresponding to 3.6{plus minus}0.6 % of granule cells across mice": a histogram of the number or percent of puff-selective cells per mice would be informative.

We added histograms showing the absolute and relative numbers of active GCs per animal (Fig. 1-1K,L).

4) How are the "1583 GCs" mentioned several times in the section defined? Are they active GCs or does that number include silent GCs (anatomically defined ROIs)? Reporting the proportion of active cells per mice (and their transient rate, to compare with past studies) would also be useful, to assess the validity of the study and check for differences in sample sizes across mice. Moreover, a histogram of the proportion of active GCs (and the number for each mouse), and of those active GCs the proportion of puff-responsive GCs would be a great illustration of what seems the driving point here: few GCs are active and even fewer encode the airpuff.

We thank the reviewer for pointing out this confusion. We used a constrained nonnegative matrix factorization-based algorithm to identify active granule cells in each FOV (Pnevmatikakis et al., 2016). This algorithm is dependent on a changing fluorescence signal to identify putative cells. In addition, we decided to include only granule cells into our analysis that show at least one significant calcium transient in their activity profile. By adding this criterion, we ensure that every cell included in this study is indeed a granule cell that was imaged with a sufficient SNR to draw conclusions about its activity. Excluding silent cells is of course a loss of one potential variable, but the estimation of the fraction of silent cells is very difficult and prone to systematic errors due to the density of the GC cell layer (even though expression of the Thy1 mouse line is sparsened). We added further explanation to this approach both in the methods as well as in the results section. A histogram showing the number of identified granule cells in each mouse was added (Fig. 1-1A).

5) Line 61 "only 54% of stimuli elicited a significant population response compared to baseline (Fig. 1H, n=9 mice, n=52 cells)." A) 54% is also the percentage of airpuffs that triggered running. Is that a coincidence? B) I don't see how Fig 1H illustrates the sentence. It does not seem like 1H is a trial-wise analysis. Moreover, I did not see any population analysis in figure 1. But it would indeed be interesting to see if the GC population, as a whole, reliably encodes the puff (using a mouse-wise decoder or manifold analysis) and whether it is more reliable than individual responses.

We added a stacked bar graph to clarify this point (Fig. 2-1A). This bar graph shows which fraction of air puffs triggered either locomotion, a population response, or both in a mouse-wise manner to further illustrate the individual differences between animals. This plot shows that there is indeed not a 1:1 correlation between the two responses.

We performed ROC analysis to test how the average of the whole population, the responding (AP+) granule cells and the other (AP-) granule cells can decode the airpuff (Fig. 1Q and Fig. 1-1O,P for mouse wise curves). This analysis revealed that the air puff could indeed not be decoded from the mean of all cells, but could be decoded from the responding AP+ granule cells (AUC Above 95th percentile) and also weakly from the other AP- granule cells (AUC below 5th percentile). This illustrates how the dentate gyrus as a population seems to remain at a stable activity level and how responses only become evident after the separation of responding and other granule cells. The AUC of the ensemble ROC analysis is correlated with the absolute number of responding GCs found in each recording (r=0.79, p = 0.01, Fig.1-1Q).

6) Fig 1D (pupil size): I do not distrust the conclusion (it is visually obvious in Fig 1B that air puffs often trigger running and pupil constriction) but in C and D, the shuffle 95% CI looks suspiciously thin to be meaningful. It is different from almost all parts of the yellow curves, even parts that should be back to baseline, whereas I would expect the variance in the shuffle to be similar to the variance in the baseline. In general, the shuffle procedures used throughout the paper could be more detailed so that we can understand what was shuffled exactly and how.

We thank the reviewer for spotting this error. There was a numerical error in the std of the shuffled data which is corrected now. We also added information on the general shuffling procedure in the methods section. In short, data snippets from random times when the animal is resting equal to the number of air puffs are picked (without repetition). This explains the negative trendline of the pupil, since the pupil is more often contracting during rest than it is dilating (Pofahl et al., 2021; Reimer et al., 2014; Reimer et al., 2016). Hence, for the longer time course it makes also a bigger difference if the mouse initiates locomotion or not (Fig.2 E-G). To emphasize the point that the quick pupil contraction serves as a good classifier for the air puff we added roc analysis (Fig. 1E).

7) I find Supplementary Fig S1A-C useful and deserving of being in the main figures. Y-axis labels reminding what each signal is would help the reader, but it conveys clearly that airpuffs, and not running, lead to activation in previously determined puff-responsive GCs. Interestingly, the average puff GCs response have a sharper signal (shorter decay) for APs without run than APs with run, suggesting subtle influences of running on GCs activity, perhaps inducing a longer decay in GC activity. Cumulative probability plots (S1D-E) are not as convincing: distributions all look highly similar. If the post-hoc tests are to be trusted, the effect size seems quite small. I find it difficult to reconcile A-C and Fig S1D. How was "response probability" calculated? And what about using different response variables, like signal amplitude, or decay time constant...? Also, the number of trials for each category should be stated somewhere in the figure. Differences in sampling might be an issue for stats and interpretation. If there are strong sampling differences (or if the sample size is too large, like for the pool of non-responsive GCs), subsampled confidence estimation statistical methods would be more appropriate than classical hypothesis testing (because sample size affects p-values).

Overall, I think additional analyses to strengthen the conclusions are warranted. For instance using GLMs or Linear Mixed Models approaches (see comment #1) to take mouse-wise variability in account and avoid relying on arbitrary thresholds to predefine puff-responsive and non-responsive GCs (see comment #2).

We added the former supplementary figure as a main figure to the manuscript (Fig. 2). The reviewer is correct that the time courses are different depending on whether running was initiated or not, and we added analysis demonstrating this point (Fig. 2E-G). This analysis shows that the excitatory response is indeed sharper when the animal remains at rest while the time course of the inhibitory response is similar in both scenarios. We believe that this may be due to an increased and prolonged MPP input when running is initiated (See point 8 and also Pofahl et al 2021) rather than by a difference in air puff response, since the excitatory response precedes the running onset.

We further added information illustrating to which extent the responding ensemble and individual GCs are activated correlated to running initiation. A mouse-wise analysis of how many air puffs triggered running, an ensemble response, or both, illustrates the differences between individual animals and that there is no 1-1 correlation in mice that had running and non-running responses (Fig. 2-1A). This difference between mice is also prevalent when looking at the individual granule cell responders per animal (Fig. 2-1B-E). Hence, to ask whether a responding granule cell is correlated to running initiation makes more sense in mice where there is a balance between locomotion and non-locomotion response. Therefore, we did this analysis in mice in which granule cell ensemble response was combined with and without locomotion initiation after airpuff presentations. In those animals we find no preference whether cells respond correlated to running initiation (Fig. 2-1D,E).

We think the former cumulative plots looking at the response amplitudes (In units of sigma of the baseline of each distribution) are indeed misleading. This is mainly because of the high number of zero responses due to sparsity even in the averaged ensemble response in both groups. We have re-done the plots using swarmcharts (Fig. 2J,K). The sample sizes are stimulation with running initiation, without running initiation and spontaneous running onset are 199, 284, 297 respectively and are stated now in the main text and the figure legend. We consider the samples balanced enough for non-parametric testing. Again, the zero values can be misleading here. However, with the new responder definition we find that the responding granule cells response is comparable between running and non-running conditions (Kruskal-Wallis test p<0.01, Dunn-Sidak post-test air puff with run-init. vs. air puff w/o run-init. p=0.9). The difference between both air puff conditions and the pure running onset is significant (air puff with run-init. vs. run onset p<0.01, air puff w/o run-init vs. run onset p<0.05). For the inhibitory response we find the amplitude be significantly lower only in the non running initiated case (Kruskal-Wallis test p<0.0001, Dunn-Sidak post-test air puff with run-init. vs. air puff w/o run-init. p<0.05, run onset vs. air puff w/o run-init. p<0.0001). This change to our initial results can be explained by more granule cells being classified as non-responder due to the new definition and systematically by the extra excitation coming in when the animal starts running.

8) Fig S1A-C also gives useful information for the MPP signal, and it looks quite different for each category, which weakens the conclusions of the authors, as they argue that MPP and GC responses are correlated but they find GC responses are similar for APs with and without running whereas it is not the case for the MPP signal. Running seems to affect MPP signal (the later part of the response especially) more than puff-responsive GCs. Relatedly, could the long-lasting MPP activation during running explain the long-timescale of the inhibition of non-responsive GCs? We agree with the reviewer and included the panels into the main figure (Fig. 3B-D). Indeed, the MPP signal looks different in all three scenarios, being more distinct when no running is initiated. The longer lasting activation can be explained by the general activation of the MPP correlated to running as we have also shown in former studies (Fig. 3D, cite Pofahl et al 2021). However, the sharp onset of the signal seems to be unique to the air puff stimulation. We found that the inhibitory response follows the same time course in both scenarios (Fig. 2I,N). In contrast to that we find for the excitatory response a even longer FWHM when running is initiated compared to a sharp response when it is not. This suggests that the running related MPP activation could drive this longer lasting activation of the responding granule cells.

9) Line 164 "The inhibitory response of the non-responding granule cells did not result in a significant signal correlation. This is likely because inhibitory responses are subtle given the overall sparseness of granule cells (Fig. 2D, E).": Isn't it just because signals were not normalized? The inhibitory response of non-responsive cell is much lower in amplitude and so will mathematically lower correlations if there's no normalization. Similarly, it does not seem appropriate to compare the 2 crosscorrelations in Fig 2E because of this lack of normalization.

We see the reviewer's point. The reason why the cross correlations were small in amplitude is because we averaged over all the individual responses of all granule cells. Those signals were of course normalized but sometimes too sparse to create a meaningful cross correlation others were averaged out. To make our point in this section clearer we changed the way we show the different correlations. To overcome the sparseness issue, we report the correlation between the df/f traces instead of the deconvolved data. We then show the cross correlations of the mean signals before we report the correlation coefficients for the individual granule cells. And as a final part we report the noise correlations between the MPP input and the granule cell signals.

10) Line 170 and 191 "individual responder granule cells (dark blue box, n = 3 mice, n = 6 cells), and for non-responding granule cells (light blue box), Kruskal-Wallis test, n = 3 mice, n = 263 cells,": There is such an imbalance in sample sizes (6 vs 263) that I think even parametric tests might not be robust and thuis not trustworthy in that case. The variance in the responder group is necessarily wrong because of under-sampling, and the variance in non-responder is huge... Moreover, one of the sample size is very high, which artificially lowers p-values and makes hypothesis testing inappropriate (for high sample sizes, resampling approaches are best suited). A Monte-Carlo subsampling approach (so that the average for the non-responsive group is sampled for each bootstrap based on 6 cells in both groups) would be more appropriate. See for instance Ho, J., Tumkaya, T., Aryal, S., Choi, H. &Claridge-Chang, A. Moving beyond P values: data analysis with estimation graphics. Nat. Methods 16, 565-566 (2019) and Dong and Sheffield (2021) for an example of subsampled bootstrap.

The reviewer is right and we are grateful for this critique. We followed the reviewers suggestion and added subsampling tests.

11) Line 194 "sensory stimulation elicits strong and wide-spread inhibition": at this stage, Fig 2 did not show that MPP correlates with the inhibition, so the statement is not supported yet. This may change once signals are normalized (see comment 9).

The reviewer is correct. We changed the way of presenting the cross correlations (See point 9) and in our opinion the statement is now supported.

12) Line 265-7: I find the claim that I/E ratio is stable, for a given frequency but also across input frequencies, not well supported by the data and inconsistent with past research that has shown DG acts as a frequency filter of EC inputs and that recruitment of inhibition depends on input frequency (e.g. Ewell and Jones 2010, Scullin and Partridge 2012, Pardi, Schinder and Marin-Burgin 2015, Hsu 2007, Lee and Lien 2016,...). This discrepancy should at least be discussed.

I find the evidence provided underwhelming for several reasons: a) the I/E ratio was not directly measured but estimated from current-clamp recordings and a model fitted to the data to estimate inhibitory and excitatory conductances. That is perfectly ok, especially for in-vivo recordings, but model fitting can be inaccurate and imprecise. The authors should present analysis of the goodness of fit of the model, and discuss the potential for parameters degeneracy, because both of these issues could explain some of the variability in their plots (errorbars are sometimes quite large. The average may appear stable across multiple stimulations but dynamics could be hidden under the noise). b) Contrarily to what is claimed (line 341 in discussion), the estimates of the i/e ratio from the slice prep (Fig S3) do not match past studies well (Ewell and Jones 2010 Fig 4, Marin-Burgin and Schinder 2012 Fig S8) as they showed a ratio between 1 and 10 (i.e. inhibition always larger than excitation) so closer to what the authors measured in-vivo. This undermines either the slice experiment or the validity of the conductance estimation model. It also undermines the claim that slice experiments lead to radically different results from in-vivo recordings (line 295). c) In contrast to the claim that i/e ratio stays stable due to similar levels of depression for I and E, Fig 4B clearly shows that the depression does not follow the same dynamics for EPSGs and IPSGs. Is the example not representative? Because it does not match with the average shown in D or F. I had similar issues reconciling the examples, normalized EPSG and IPSGs plots and the I/E ratio plots in Fig S3. For example, in the 20Hz and 30Hz conditions, the examples suggest that the I/E ratio should quickly rise above 1 after the first PSG, but that is not the case in the average i/e ratio. Again, this may be due to added noise from the estimation model leading to large variability across cells. Relatedly, the imprecise estimation may also explain why individual PSGs are not clearly observed in the 30Hz condition, in contrast to what is usually observed in actual recordings from GCs (e.g. Ewell and Jones 2010). Unless this is due to using optogenetic stimulation rather than electrical stimulation as in past studies (this should be discussed). d) Finally, the claim that I/E is stable is based on Friedman tests (non-parametric repeated-measure anovas) that may not have been powerful enough to detect differences. The doubt is especially high for Fig 4F where the I/E ratio is noisy and the p-value was close to significance (0.16). Again, the example shown in B is a direct counter-example to the claim for stability. Furthermore, the lack of detected effect is not the same as the absence of effect. To rigorously claim that I/E was stable across stimulations, an Equivalence Test would be needed.

We thank the reviewer for their detailed analysis of our findings regarding the I/E ratio and its stability during frequency stimulation. First, regarding the I/E ratio, we find that in our in-vitro preparation the excitation is much larger than the inhibition and the reviewer is correct, this is in disagreement with previous studies. Ewell &Jones (Ewell and Jones, 2010) find IPSCs about 3x larger than EPSCs and Marín-Burgin et al. (Marín-Burgin et al., 2012) (2012) find them to be about twice as large. Comparing their and our methodology the only plausible explanation for the discrepancy we found is that we isolate the currents with gabazine, while Ewell &Jones and Marín-Burgin et al. rely on voltage clamping to the respective reversal potentials. Our gabazine wash-in shown in Figure S2 D shows that even when clamping at 0mV, the inhibitory current is masked by an excitatory current and the excitatory current is masked by an inhibitory current even at -80mV. But gabazine increases the EPSC more than the subtracted IPSC. This leads us to conclude that if a gabazine based subtraction method is not used, both EPSC and IPSC magnitudes may be underestimated, but the EPSC is likely underestimated by a larger extent. Other than this, we could not find reasonable explanations for the discrepancy. Our conductance estimation in-vitro is very simple, relying on voltage clamp and Ohm's Law and because of the values of the voltage clamp changes only the magnitude but not the I/E ratio.

Second, regarding the stability of I/E ratio, our conclusion is based on the lack of a significant effect of stimulus # on I/E ratio. The reviewer is justified to question this conclusion, because we might be making a statistical beta-error, that is concluding non-significance although in reality there is a significant change. An equivalence tests only changes the direction of the statistical error but we can perform a post-hoc power-analysis to find out how likely it is that we erroneously miss I/E dynamics. The power analysis of the Friedman test we used showed low power (0.082 for 5Hz and 0.33 for 20Hz). This indicates that there is a high chance that we are missing real I/E dynamics. This is most likely due to the low number of samples and the large variability. We now report, along with the results of the statistical test, the caveat of the low power, which is indeed a limitation both in the results and in the discussion.

13) Line 341: I cast my doubts above on the claim that slice experiments yield radically different results from in-vivo experiments. The authors should at least discuss what could cause the discrepancy between their in-vivo and in-vitro results. Is it, as I suggested, mostly a reflection of inaccuracies in the estimation model? Or is it real, and in that case, is it due to different parts of the hippocampus (dorsal for in-vivo vs ventral for in-vitro?)? temperature? Age of mice? GC maturity? Slice angle limiting some inhibitory projections? ... These details should be provided in the methods for comparison with past studies.

We have amended the methods to include more detailed descriptions of the methods, and have amended our discussion of the discrepancy both with published in-vitro data and the in-vivo findings.

Minor comments Line 16 "inhibition is thought to critically contribute to the function of pat sep [refs]": Many of these references do not study inhibition and most, if not all of them, conflate mnemonic discrimination (behavioral pattern separation) with the neuronal operation called "pattern separation". Please clarify what you mean by pattern separation, and if you mean the neuronal operation, I would at least add some references directly supporting the statement, like O'Reilly and McClelland 1994, Madar et al. 2019 and Braganza 2020 (which the authors cite elsewhere).

We thank the reviewer for that suggestion. We added the citations.

Line 18-25: the paragraph motivating the study is quite vague, without a clear question. The literature on recruitment of inhibition in the dentate and how critical it is for gating EC inputs and performing pattern separation, including in-vivo work (e.g. most recently Hainmueller and Bartos 2024), is deep and hardly mentioned. The authors could identify more clearly the knowledge gaps.

We thank the reviewer for identifying that gap. We have revised the introduction to include a clearer picture of the recruitment of interneurons in the dentate gyrus, focusing on in-vivo studies. We have also made clearer the knowledge gap and research question that our study addresses.

Line 22 "intrahippocampal connectivity not encompassed by slice prep": Are the authors referring to mossy cells projections? The Bienkowski 2018 ref did not study EC projections to DG, as far as I understand. Refs like Yen, Monyer and Lien 2022 (interhemispheric DG SOM projections), may be more relevant to the statement.

We thank the reviewer for this suggestion and added the suggested citation.

Line 40 "mice ran on a linear track": More details on the experimental set-up and mouse behavior is needed, both in the result and method section. For instance, were mice running on a wheel navigating a virtual linear track displayed on a screen or are they running on a belt? What are the sensory cues? Is it spontaneous running or are there some elements of training or implicit learning? I surmise that mice are running on a belt and are mostly motivated to run by the air puffs, but this does not appear explicitly stated anywhere.

We added more details on the experimental paradigm and the running belt in the results and the methods section.

Line 44: similarly, we need more details on the air puff implementation. How often were they presented? Was it random? Was it at every pause? Was some learning involved? What were the parameters conditioning the occurrence of an airpuff? The method section could also give a lot more details on how air puffs were delivered, where on the body, the intensity, etc..

We added more details and statistics on the presentations of air puff stimuli in the results and the methods section (Fig. 1-1 C-D).

Line 44 "54% of trials (Fig 1C n = 10 mice)": the sample size should be a number of trials. Moreover, The number of mice is inconsistent with the figure legend which says n = 9. Same problems for Line 46 " 87% of stim presentations (Fig 1D n = 10 mice)".

Thank you for noticing this - we changed the n to the number of stimuli where it was relevant.

Line 50 "low activity levels which can vary over a wide range": Danielson and Losonczy/Kheirbek 2016 should be added to the references.

We added this citation.

Line 52 "we analyzed each cells activity": each cell We corrected this error.

Line 62 "(Fig. 1H, n=9 mice, n=52 cells).": n cannot have 2 different values. Both numbers are important (but perhaps do not need to be repeated all the time throughout the text) but only one correspond to the sample size. The other is the number of replicates if cells from all mice were pooled in the analysis. Same issue line 170, 190-1 and elsewhere.

We changed all information for n to just the relevant one in each case.

Line 65 "We found that each granule cell on average responded to 25% percent of stimuli where the peak of this distribution is at 12.5% suggesting that most granule cells respond to a smaller number (Fig. 1I, n=9 mice, n=52 cells).": The syntax makes the sentence unclear.

We changed this sentence.

Fig 1E-F: why are E and F separated? Do they represent 2 different responsive GCs (as I surmise) or do each plot combines a bunch of responsive GCs (as the title in E suggests). Please clarify in the panels and legends.

We specified the figure legend accordingly.

Fig 1J: y-axis label is misspelled For many plots, such as the x-axis in Fig 1J, labels are just units or lack a clear description. For clarity, I encourage the authors to add meaningful labels or titles (e.g. in Fig 1L, P, Q) in the panels directly, so that we can know at a glance what is plotted.

We added labels and titles to the panels.

Fig 1K: how is onset measured? On the average response, like the ones plotted in 1G? Line 110 legend of Fig 1H: Please clarify how "onset probability" is calculated. Is this really a probability between 0 and 1, as it is expressed in units of variance? Or is it the average of traces in G? This is the z-scored amplitude in units of variance. We corrected that and tried to be more precise and consistent.

Line 127: The legend of Fig 1Q is unclear. Please specify dynamics of what. Not sure what is meant by "in-activation".

We changed the caption of this panel.

Line 141 and 146: Why is "AIR PUFF" capitalized here? This was a formatting error and has been corrected.

Line 167 and line 189 "noise correlation": This is a confusing term here. Noise correlation usually refers to the correlation between multiple repetitions to assess variability in a neuron's response to the same stim, not between 2 different kinds of signal (MPP and GCs). Did you just mean it was a trial-wise analysis, without any lag, as opposed to Fig 2E which was correlating averaged signals and varying their lag? Please provide more details here and in methods on what exactly was correlated, both for Fig 2E and 2F. I did not find the method section very clear.

The reviewer is correct that noise correlation describes the correlation of two neurons in a network when the mean response to a repeated stimulus is subtracted (Averbeck et al., 2006; Hazon et al., 2022; Meir et al., 2018). In our case, the airpuff is a repeated stimulus and we are interested if the response fluctuations (the noise) of individual granule cells correlate with the stimulus response fluctuations of the MPP input. We agree that we stretch the original definition by looking at two different synaptically coupled elements in the network, but the question we ask is answered by the concept of noise correlation and we would therefore like to keep this term. We also clarified our noise correlation approach in the methods.

Fig 2C-E legends: please specify what all errorbars represent.

Done.

Line 206 "MEC principal cells were stimulated": Please specify here whether it was MEC axons in DG or MEC somas.

Done.

Line 217 "To isolate excitatory (ge) and inhibitory conductances (gi), we maintained the membrane potential of granule cells to four different levels using constant current injection.": This is a somewhat confusing phrasing because recordings are in current-clamp mode, so the membrane potential is not what is "maintained" per se.

The reviewer is correct, we rephrased the sentence.

Legend Fig 3C "Close up off": of Thanks. Typo has been corrected.

Legend Fig 3I "Delay from stimulation time of excitatory and inhibitory to conductance response onset as well as the delay between both onsets (black).": syntax makes it confusing We changed the syntax of the two legends.

Fig 3F: was the I/E ratio computed on normalized PSGs like in Fig 4 or S3? The power is a poor indicator of input strength, given how variable the number of recruited fibers can be from one animal to the next (depending on expression, focus of the stim, depth...). Hence some large errorbars maybe. What about plotting things as a function of the size of the first EPSG (or as a function of the amplitude of the first EPSP under gabazine when this is available, for in-vitro data) to better estimate the level of recruitment of the MPP? The reviewer is correct that the laser power is not perfect to estimate the input strength. We tried other normalization approaches as suggested by the reviewer, which did not lead to improvements of clarity. Since we are aware that the estimated values are difficult to compare between animals, we did all frequency analysis at saturated responses which were comparable between animals.

Fig 4C-F and Fig S3: please specify what errorbars represent Done.

Legend Fig 4C-D: what is the amplitude normalized to in C and D? First PSG? That is correct. We added this information.

Line 286 "the responses saturated at light powers at the light fiber front aperture comparable to the in-vivo experiments": syntax unclear We changed the syntax of this sentence.

Fig S2: legends for panels E and D have been inverted.

We corrected that error.

Fig S2: no quantification is shown for the gabazine experiment.

Line 330-332 "k-winners take all" references: O'Reilly and McClelland 1994 The citation has been added.

Line 339 "It is off note that": of The typo has been corrected.

Method section Sex of mice? Both sexes were used. We added this information and the exact numbers. correlation analysis "scorr": Isn't the function used xcorr instead? Yes, that is correct. We corrected the typo. "a multimode fiber was implanted through the craniotomy of the virus injection under an angle of 15{degree sign}.": The location of that sentence, way before the description of the craniotomy itself, is surprising. Angle of 15 degrees with respect to what? more details on location of fiber needed (cranial coordinates? In DG or MEC?) We also need more details on intensity, pulse duration and input frequency of the stimulation.

We clarified this section.

For the patch-clamp experiments, more details are needed on temperature (for in-vitro), longitudinal and proximodistal axes locations of recordings, resistance and capacitance of recorded GCs (needed to make sure the population is made of homogeneous mature GCs). Given some evidence for differences between blades, were the 2 blades sampled similarly? Reviewer 2 In this study, the authors take advantage of the use of air puffs as sensory stimuli to demonstrate that medial performant path inputs can lead to activation of a small number of granule cells while simultaneously reducing the activity of the remaining granule cell population. The authors raise interesting questions by examining the balance between inhibition and excitation within the dentate gyrus circuitry and by discussing the importance of their findings for the function of pattern separation. However, I have some concerns that would need addressing.

Specific comments:

1. " A granule cell was defined as a responder if it showed significant Ca2+ transient onsets following at least four air puff stimuli and if this activity was shown to be larger than activity in the inter-trial interval by shuffling analysis (see Methods, Fig. 1E, F)." Four air puff among how many? This would be reasonable to express this as a fraction of the administrated stimuli. In the methods, the authors indicate "Per session at least 60 stimuli were administered". Would that mean that a granule cell reacting to air puff in 4/60th occasion would be considered an air puff responding cell? How has this number been decided for? Figure 1I also seems to show that most of the granule cells identified as responding to the air puff are actually responding to less than 20 % of the air puffs. Also, how do you reconcile this low reliability in the response to the air puff with the finding that "medial perforant path-triggered inhibition is large, fast, and maintained during repetitive stimulation"? We thank the reviewer for pointing that out. We hard coded threshold was not very elegant and mainly thought as a way to exclude granule cells of very low activity, whose response could still be significant compared to a shuffled distribution. We changed the threshold procedure to be adjusted to fixed percentage of airpuffs (e.g. 5%). However, following the suggestions of reviewer 1 we implemented various other approaches to define responders (See reviewer 1 point 2). After comparing these, we decided to switch our responder detection to a ROC analysis, since it is the most robust definition when it comes to low activity cells not requiring an additional threshold. The comparison of the different methods can be found in Reviewer Fig. 1. We plotted the overlap of the different methods with and without an additional threshold. Further, we produced some of the main panels of the study using the different definitions (Reviewer Fig. 1C-J). This analysis revealed that our main findings are robust for all different definitions.

2. In 9 mice, the authors imaged 1583 granule cells, but also state that they recorded the activity of up to 500 granule cells per FoV. Please indicate the average number of granule cells recorded per dataset (e.g. 176 +/- SEM) instead of the maximal number achieved in a rather exceptional dataset.

The reviewer is correct. We changed how we presented and added a panel showing the number of active granule cells per mouse (Fig. 1-1A) 3. Figure 1N: this cell seems to be often active around the 3 sec mark after the air puff, so much that it doesn't look random. Did the authors observed delayed responses? Did they take a look at the granule cells activity over more than 1 sec after the air puff (e.g. 5 or 10 sec)? We tested the granule cells in different response windows for later activation or rebound effects. We were not able to find any systematic late responses.

4. Figure 2: The example FoV is not sufficient to make clear that the MEC inputs labeled with jRGECO1a that are imaged (as shown in A) are the projections to the DG GCs, and not to CA3 for example. Larger images and/or post-hoc reconstruction of the area imaged would be beneficial here, as the MEC inputs terminating in CA1, CA3 and the DG are showing specific characteristics (Cholvin et al., 2021). Along the same lines, in the Methods section: "Correct injection site in the medial entorhinal cortex was verified by confined expression of jRGECO1a in the middle molecular layer of the dentate gyrus." Some histological evidence of the extent/specificity of the infection in the MEC would be a plus here.

The reviewer is correct. Since the animals were already used in a former study, the relevant histology is already published (Pofahl et al., 2021) (cite Pofahl 2021, Fig.1 suppl.1). We added this information to the methods section.

5. Methods section: "Cortical and CA1 tissue was aspirated using a blunted 27-gauge needle until the blood vessels above the dentate gyrus became visible." Damage to CA1 is associated with altered hippocampal network dynamics and disruption of the normal activity of the granule cells. In humans, hippocampal sclerosis (a pathological change long-associated with epilepsy) induces neuronal loss and gliosis centered on the CA1 subfield. Associated impacts on the dentate gyrus have been identified, including dispersion of the granule cell layer (Houser, 1990), sprouting of the mossy fiber axons (Sutula et al., 1989) as well as changes to hippocampal interneurons, including dentate gyrus interneurons (Maglóczky et al., 2000). In mice, it has also been shown that interneurons with cell bodies in CA1 constitute a numerically significant population that relays CA activity back to granule cells (Szabo et al., 2017), and that these boundary-crossing interneurons provide retrograde channeling of SWR-related activity from the downstream CA areas back to the DG. Altogether, this should be made clear, and probably discussed accordingly, that the removal of CA1 is not without consequences, especially when studying the hippocampal circuitry.

We acknowledge that - even though we kept the aspirated volume to an absolute minimum - the aspiration of CA1 overlaying the DG might lead to altered dentate gyrus activity. We now specifically acknowledge this in the methods, in particular the possibility that we might impair interneurons projecting from CA1 in to dentate gyrus (Szabo et al., 2017), as suggested by the reviewer.

6. Abstract: the MPP was imaged as a bundle of fibers, and not as individual axon terminals. This should be made clear in the abstract in the sentence "Dual-color imaging of both medial perforant path input fibers and granule cell activity allowed us to probe input-output conversion in this pathway." This is a very valid point and we clarified the used method in the abstract.

7. Discussion: "Our two-photon in-vivo imaging data show that on average only ~3% of dentate gyrus granule cells are activated following a sensory-evoked MPP activation". This ~3% represents the number of granule cells classified as air puff responding cells, but as they are not very reliably responding to this stimulus, the fraction of cells actually activated following each "sensory-evoked MPP activation" is much lower? That is true. We have added the analysis looking at the number of activated granule cells after each stimulus (Fig. 1H). This analysis also revealed that less granule cells are active after the airpuff stimulus compared to random other time points during rest. We have added a statement to the discussion that reflects this point.

8. Discussion: "In parallel, a majority of granule cells was reliably suppressed over a time window of up to one second." Maybe I missed it, but did the authors define criteria to selectively identify the cells that were reliably suppressed by air puffs or MPP activation? While this is perfectly clear that the authors classified 52/1583 granule cells as responders to the air puff, this is unclear how many suppressed cells have been identified. As this category (suppressed cells) would very likely not fully overlap with the non-responders, this would deserve to be clarified, and the suppressed cells to be considered as a specific category throughout the manuscript / analyses.

We thank the reviewer for pointing that out. Indeed, we do not define a separate class of suppressed cells. To address the reviewer's question, we tested whether we find cells that would show a significant suppression of activity with ROC analysis. We did not identify any cell that would match the 5th percentile threshold for an AUC < 0.5. Thus, a significant inhibitory effect only emerges when averaging over many cells and trials. I our opinion, the seemingly small effect in individual granule cells is due to the sparse activity of these cells. Visualization of the full extent of the distributed inhibitory effect requires the measurement of a large population of neurons. Therefore, even if the responses are not significant for individual cells, we believe that it is crucial to report the population effect.

To avoid confusion caused by wording, we tried to more clearly define the group of responding granule cells (AP+ cells) and the group of all other granule cells that either never or not significantly respond (AP- cells). Further, we pointed out that these two groups are not necessarily independent ensembles within the granule cell population, but rather living on a continuum where we set the border solely based on the significant responsiveness of AP+ cells.

9. Significance statement: "Our data directly elucidate how excitatory inputs evoke responses that are mainly inhibitory." This appears like a bold statement when many unknowns remain: type of interneurons involved / exact circuitry, if it applies to other excitatory inputs coming from the MEC or is rather specific to the sensory modality used here, etc. In accordance with the results presented here, it appears necessary to reconsider this formulation.

Indeed, we consider our findings worthy of a bold statement but agree with the reviewer that our data do not cover the full extent of possible mechanisms in the network investigated. Therefore, we reformulated the statement and toned it down. - References Averbeck BB, Latham PE, Pouget A. 2006. Neural correlations, population coding and computation 7:358-366. doi: 10.1038/nrn1888.

Danielson NB, Kaifosh P, Zaremba JD, Lovett-Barron M, Tsai J, Denny CA, Balough EM, Goldberg AR, Drew LJ, Hen R, Losonczy A, Kheirbek MA. 2016. Distinct Contribution of Adult-Born Hippocampal Granule Cells to Context Encoding. Neuron 90:101-112. doi: 10.1016/j.neuron.2016.02.019.

Engelhard B, Finkelstein J, Cox J, Fleming W, Jang HJ, Ornelas S, Koay SA, Thiberge SY, Daw ND, Tank DW, Witten IB. 2019. Specialized coding of sensory, motor and cognitive variables in VTA dopamine neurons 570:509-513. doi: 10.1038/s41586-019-1261-9.

Ewell LA, Jones MV. 2010. Frequency-tuned distribution of inhibition in the dentate gyrus. Journal of Neuroscience 30:12597-12607.

Hazon O, Minces VH, Tomàs DP, Ganguli S, Schnitzer MJ, Jercog PE. 2022. Noise correlations in neural ensemble activity limit the accuracy of hippocampal spatial representations 13:4276. doi: 10.1038/s41467-022-31254-y.

Marín-Burgin A, Mongiat LA, Pardi MB, Schinder AF. 2012. Unique processing during a period of high excitation/inhibition balance in adult-born neurons 335:1238-1242. doi: 10.1126/science.1214956.

Meir I, Katz Y, Lampl I. 2018. Membrane Potential Correlates of Network Decorrelation and Improved SNR by Cholinergic Activation in the Somatosensory Cortex 38:10692-10708. doi: 10.1523/JNEUROSCI.1159-18.2018.

Pilz GA, Carta S, Stauble A, Ayaz A, Jessberger S, Helmchen F. 2016. Functional Imaging of Dentate Granule Cells in the Adult Mouse Hippocampus. Journal of Neuroscience 36:7407-7414.

Pnevmatikakis EA, Soudry D, Gao Y, Machado TA, Merel J, Pfau D, Reardon T, Mu Y, Lacefield C, Yang W, Ahrens M, Bruno R, Jessell TM, Peterka DS, Yuste R, Paninski L. 2016. Simultaneous Denoising, Deconvolution, and Demixing of Calcium Imaging Data. Neuron 89:285-299. doi: 10.1016/j.neuron.2015.11.037.

Pofahl M, Nikbakht N, Haubrich AN, Nguyen TM, Masala N, Distler FJ, Braganza O, Macke JH, Ewell LA, Golcuk K, Beck H. 2021. Synchronous activity patterns in the dentate gyrus during immobility. eLife 10. doi: 10.7554/eLife.65786.

Reimer J, Froudarakis E, Cadwell CR, Yatsenko D, Denfield GH, Tolias AS. 2014. Pupil fluctuations track fast switching of cortical states during quiet wakefulness. Neuron 84:355-362. doi: 10.1016/j.neuron.2014.09.033.

Reimer J, McGinley MJ, Liu Y, Rodenkirch C, Wang Q, McCormick DA, Tolias AS. 2016. Pupil fluctuations track rapid changes in adrenergic and cholinergic activity in cortex. Nature communications 7:13289. doi: 10.1038/ncomms13289.
