# Supplementary material for: In Vivo Analysis of Medial Perforant Path-Evoked Excitation and Inhibition in Dentate Granule Cells
Source: eNeuro. 2025 Dec 9;12(12):ENEURO.0065-25.2025. doi: 10.1523/ENEURO.0065-25.2025 (PMC12697386; doi:10.1523/ENEURO.0065-25.2025)
Supplement: Data 2 — Download Analysis Code legend, DOCX file. [file eneuro-12-ENEURO.0065-25.2025-s011.docx]

Analysis code legend

Imaging:

Running the script makeFigures.m runs the analysis on the 2p imaging data and produces all panels for Figure 1,2 & 3 of the study. The panels are distributed in 5 individual files. The helpers-folder contains all functions for analysis.

Patching:

The script inexanalysis.m runs the E/I balance analysis on the data. The helpers folder contains scripts for the relevant analysis.

A GUI can be opened through schowdata.m that allows to inspect the data. The gui folder contains plotting scripts and some additional analysis.

PoolData.m pools the data of identified granule cells together
