# Supplementary figures and images for: In Vivo Analysis of Medial Perforant Path-Evoked Excitation and Inhibition in Dentate Granule Cells
Source: eNeuro. 2025 Dec 9;12(12):ENEURO.0065-25.2025. doi: 10.1523/ENEURO.0065-25.2025 (PMC12697386; doi:10.1523/ENEURO.0065-25.2025)

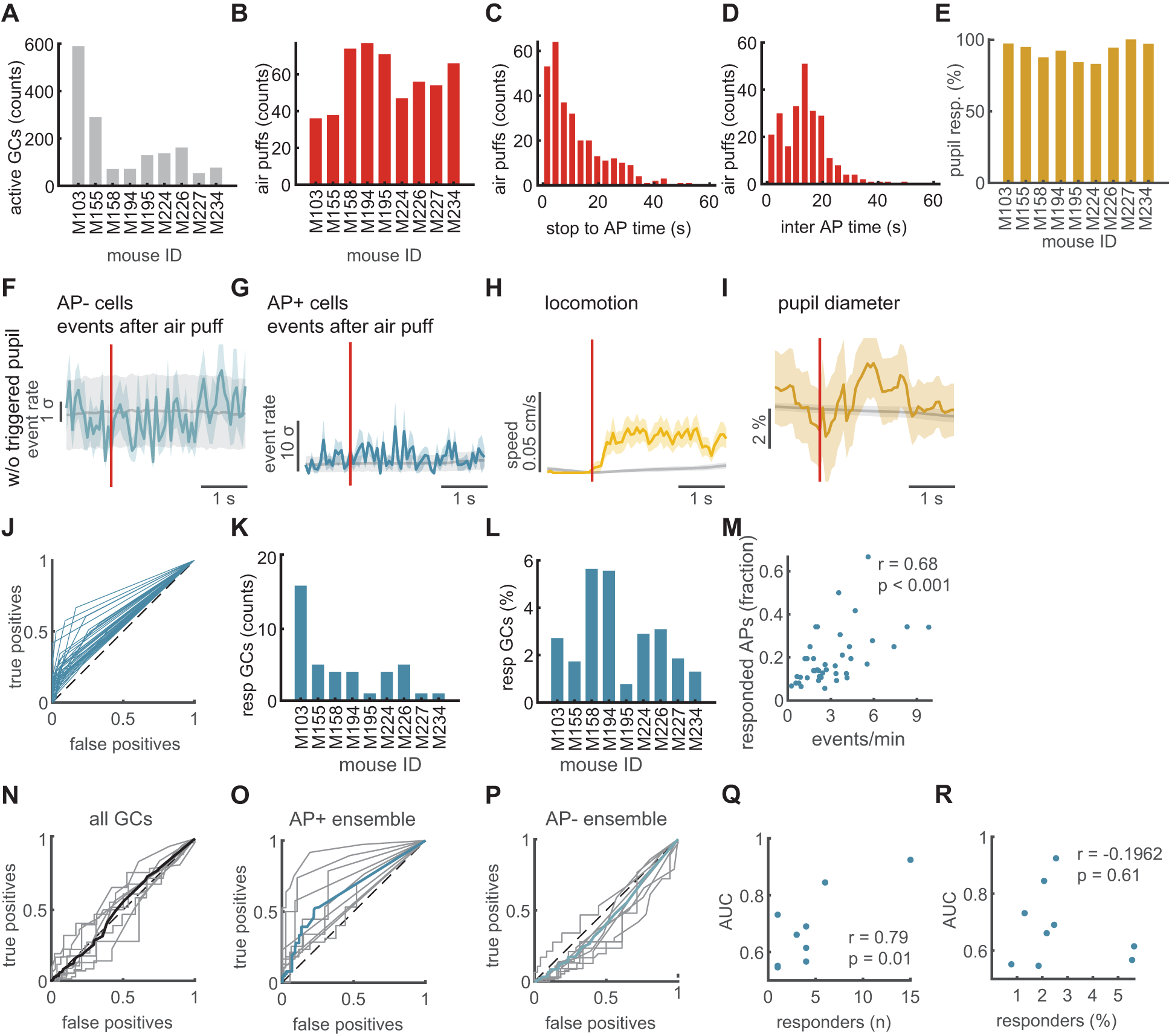

Supplement: Figure 1.1 — Figure 1-1: Imaging dentate gyrus granule cell responses during an air puff stimulation paradigm. A, Numbers of identified active granule cells for individual mice. B, Numbers of applied air puff stimuli per mouse C, Time intervals between stopping of an animal and the first air puff stimulation D, Time intervals between airpuffs E, Fraction of air puffs that triggered a significant pupil response for individual mice. F, Mean of granule cell activity after air puffs that did not trigger significant pupil dilation for all granule cells except significant responders. G, Mean of granule cell activity after air puffs that did not trigger significant pupil dilation for significantly responding granule cells. H, Mean of running speed after air puffs that did not trigger significant pupil dilation. I, Mean pupil dynamics after air puffs that did not trigger significant pupil dilation. J, ROC curves for all significantly responding granule cells K, Numbers of significantly responding granule cells for individual mice L, Fractions of significantly responding granule cells for individual mice M, Correlation of response probability of individual responding granule cells to their overall activity rate (n = 38 cell from 9 mice, r = 0.68, p < 0.001) N, ROC curves for the mean activity rate of all granule cells for individual mice (grey lines) and the pooled data set (Black line). O, like K for the mean of significantly responding granule cells P, like K for the mean of all granule cells except the responding granule cells Q, Correlation of the AUC derived from the mean signal of significantly responding granule cells from individual mice against the absolute number of responders in each FOV (n = 9 mice, r = 0.79, p = 0.01) R, (Non-) correlation of the AUC derived from the mean signal of significantly responding granule cells from individual mice against the absolute number of responders in each FOV (n = 9 mice, r = -0.2, p = 0.61). Download Figure 1.1, TIF file. [file eneuro-12-ENEURO.0065-25.2025-s001.tif]

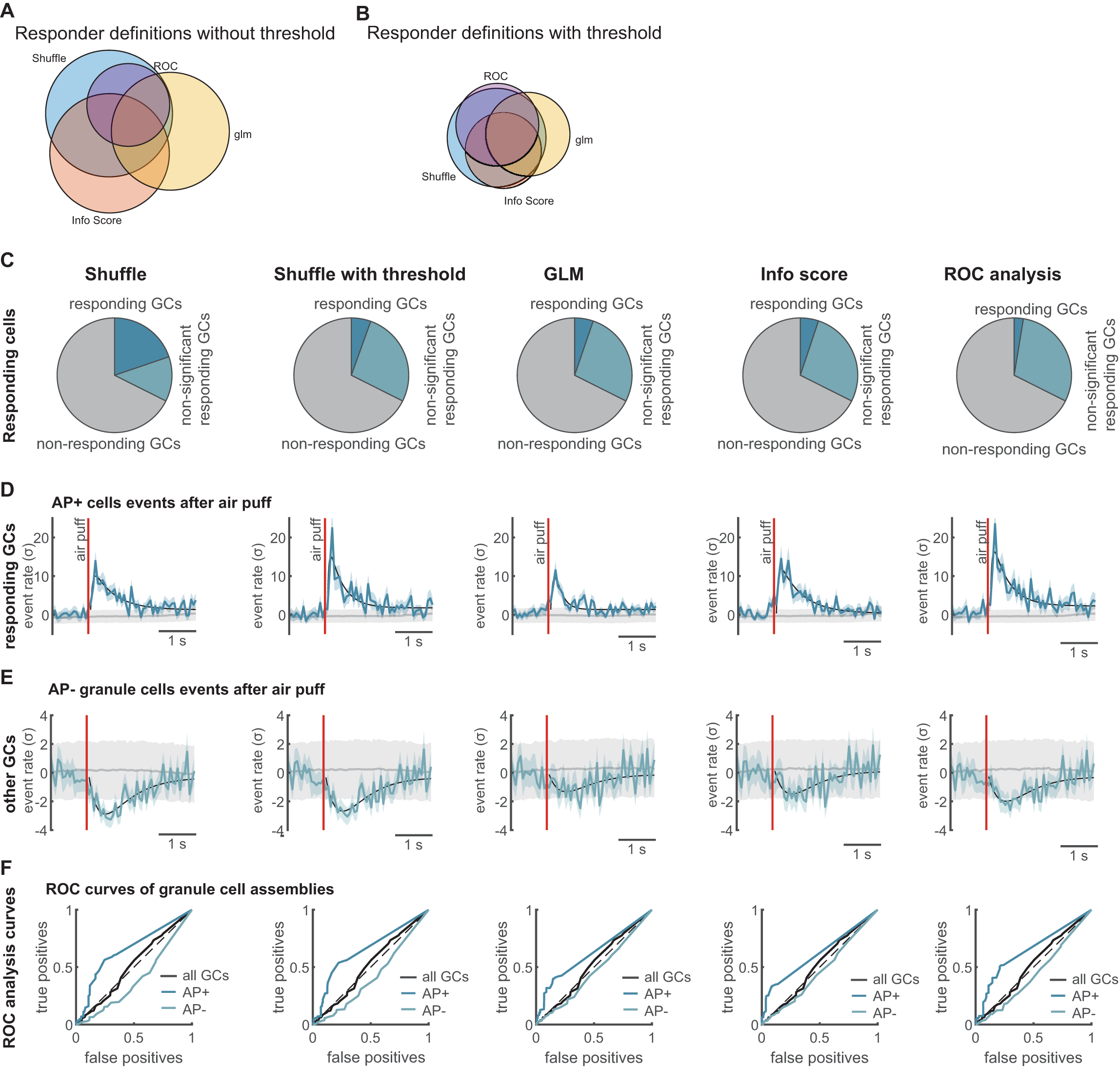

Supplement: Figure 1.2 — Effects are robust for different responder definitions A, Venn diagram illustrating the granule cells identified for the AP+ group using different responder definitions. ROC analysis (purple), shuffling approach with 95th percentile (blue), mutual information score (orange), and glm based identification (yellow). B, Like A with an additional threshold of 5% of responded stimuli per cell. C, Analogous to panel 1L for different responder definitions D, Analogous to panel 1O for different responder definitions E, Analogous to panel 1P for different responder definitions F, Analogous to panel 1Q for different responder definitions. Download Figure 1-2, TIF file. [file eneuro-12-ENEURO.0065-25.2025-s002.tif]

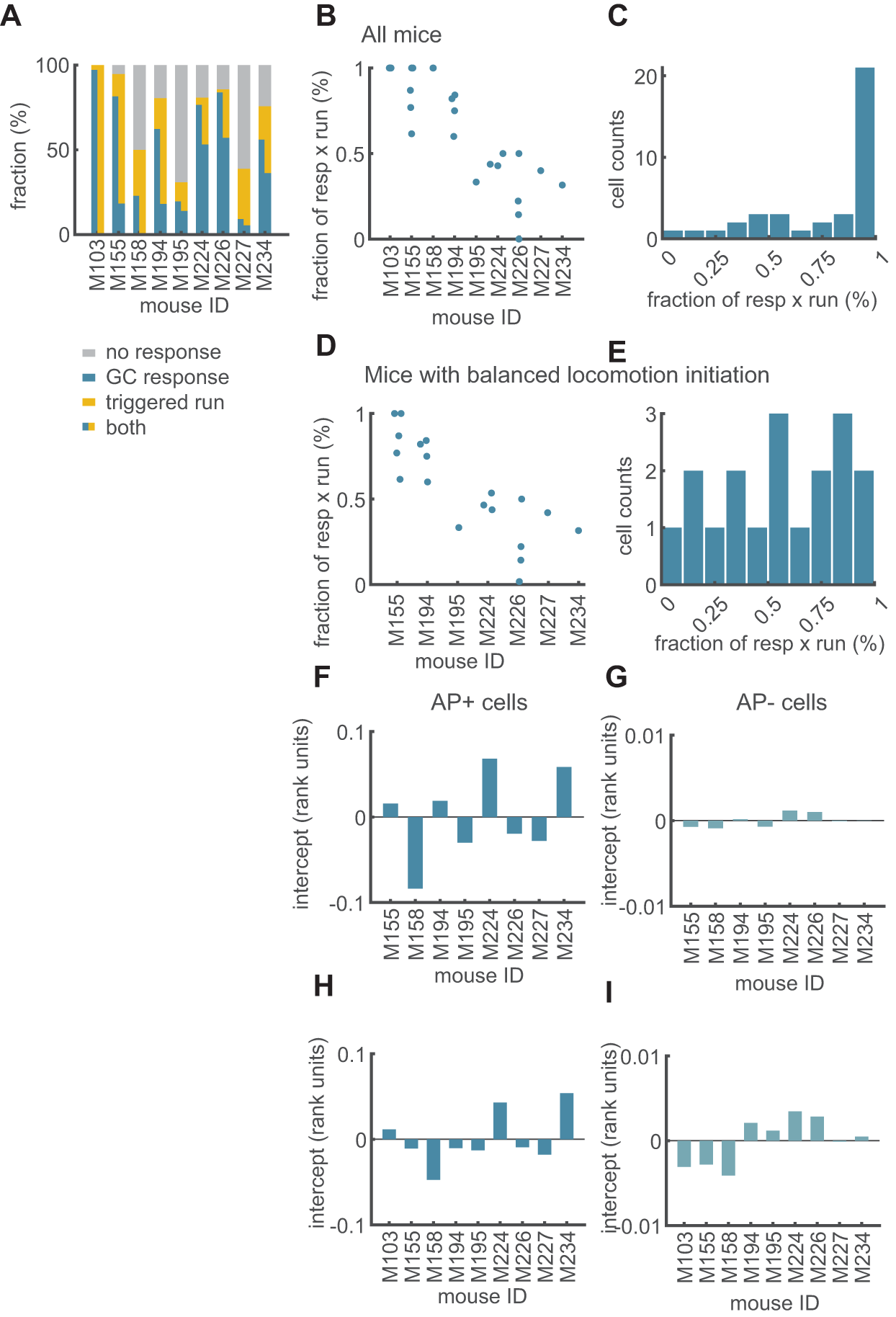

Supplement: Figure 2-1 — Granule cell responses are not correlated with triggered running initiationA, Bar graph denoting the fraction of air puffs that triggered running (solid yellow), a granule cell ensemble response (solid blue), both (blue and yellow), or neither (grey) for individual mice. B, Scatter plot showing each individual responding granule cell in each animal the fraction of responses that were combined with a triggered running initiation. C, Histogram counting the cells with a specific fraction of responses that were combined with a triggered running initiation D, same as B but only for mice that showed granule cells responses that were combined with and without a triggered running initiation. E, same as C but only for mice that showed granule cells responses that were combined with and without a triggered running initiation F, Intercepts of individual animals from the test presented in panel 2J. The overall intraclass correlation is 0.19. G, Intercepts of individual animals from the test presented in panel 2K. The overall intraclass correlation is 0.004.H, Intercepts of individual animals from the test presented in panel 2L. The overall intraclass correlation is 0.2. I, Intercepts of individual animals from the test presented in panel 2M. The overall intraclass correlation is 0.04. Download Figure 2-1, TIF file. [file eneuro-12-ENEURO.0065-25.2025-s003.tif]

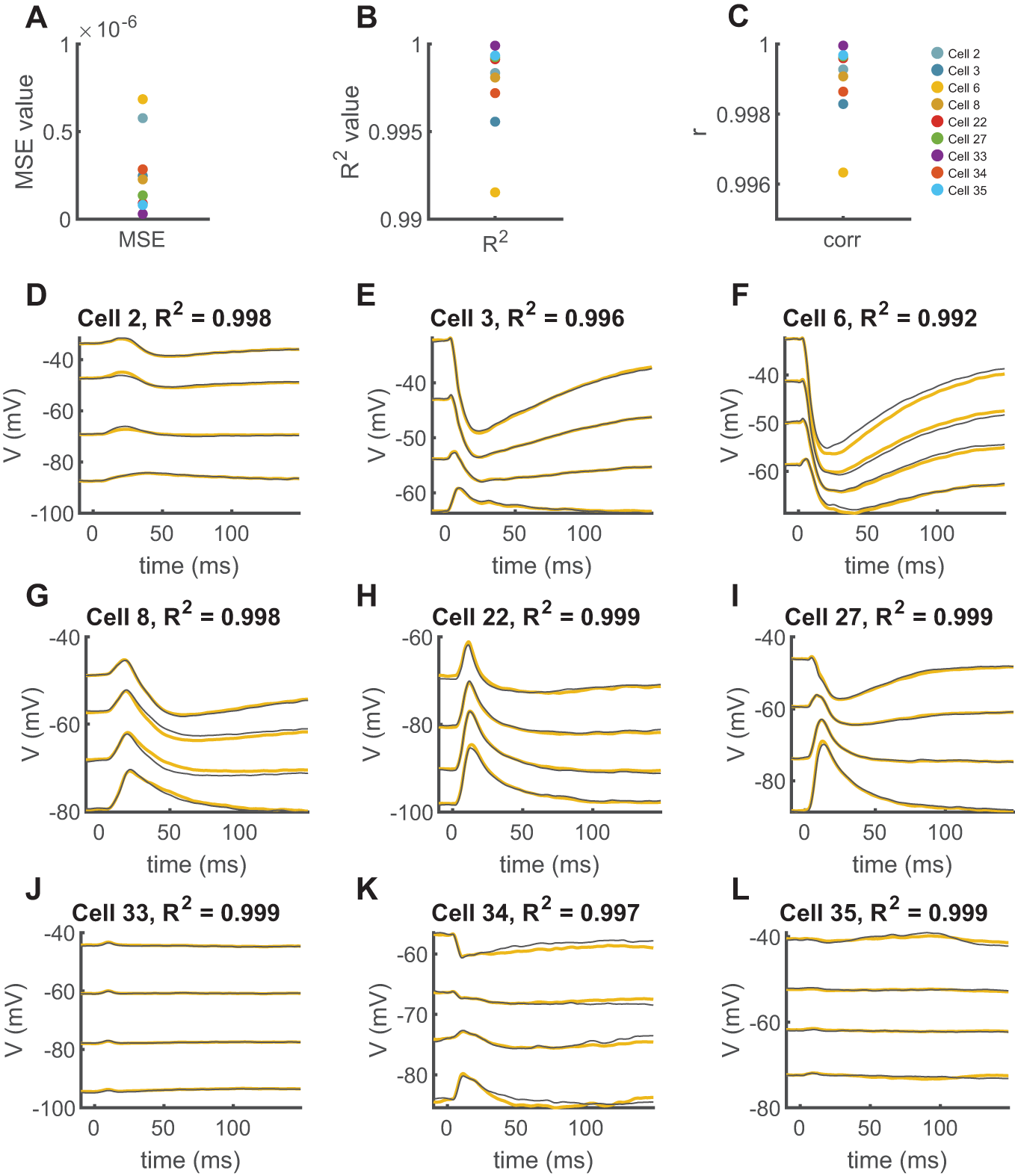

Supplement: Figure 4-1 — Error estimation of the excitation/inhibition model A, Mean squared error (MSE) of the model fit for each responding granule cell in the data set. B, R2-value of the model fit for each responding granule cell in the data set. C, Correlations between the input data and the reconstructed traces after the model fit for each responding granule cell in the data set. D-L, Example response for each patched and current clamped granule cell. Measured voltages for the four different current injections in black. Reconstructed voltage traces after the model fit in yellow. Download Figure 4-1, TIF file. [file eneuro-12-ENEURO.0065-25.2025-s004.tif]

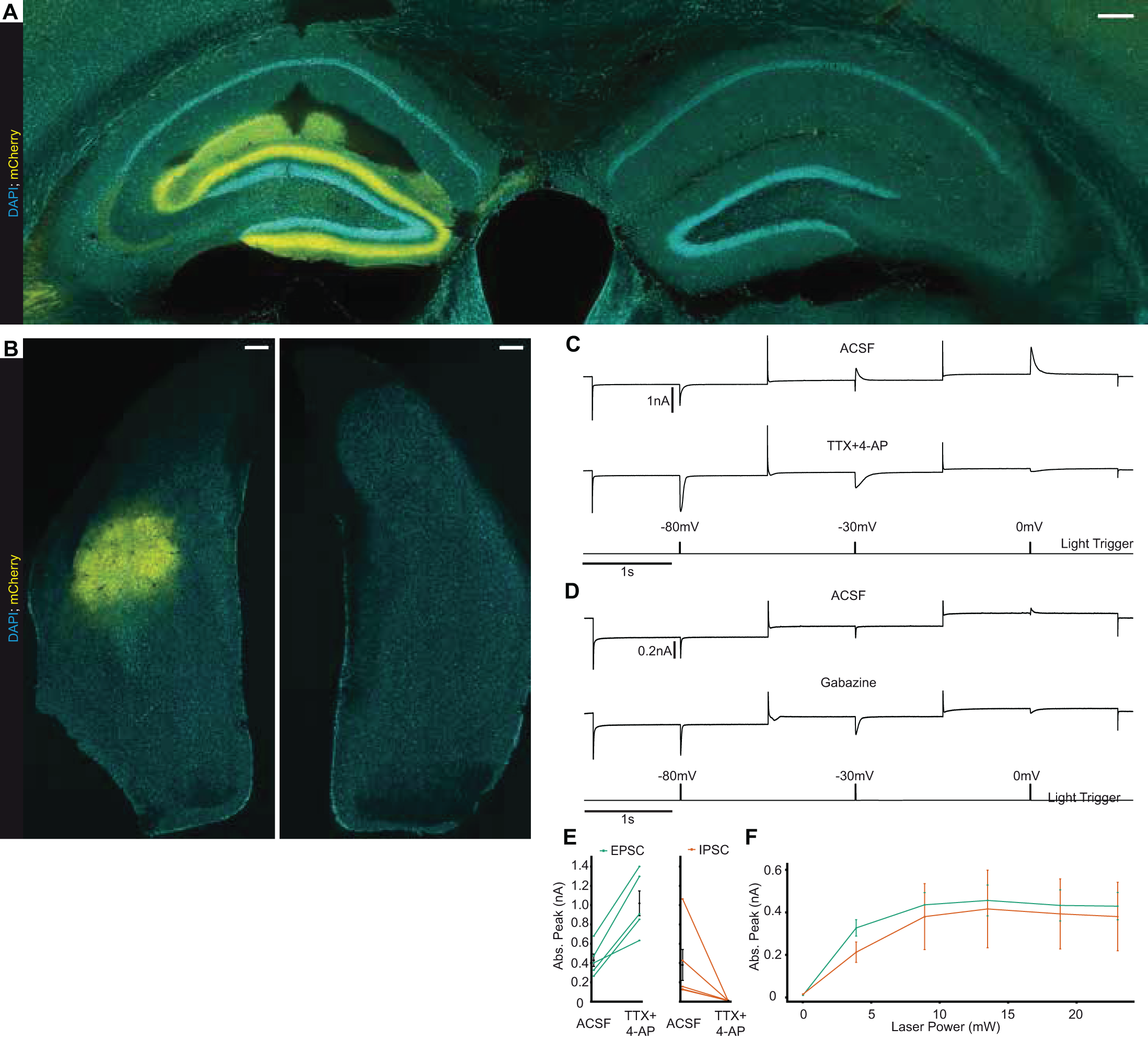

Supplement: Figure 4-2 — Optogenetic MPP stimulation elicits monosynaptic EPSCs and polysynaptic IPSCs A, Histology of acute coronal slice preparation of dorsal hippocampus. MPP fibers of left hippocampus express ChR2-mCherry (yellow), as evident from the fluorescent band in the middle molecular layer of the dentate gyrus. B, Coronal slice of MEC, showing ChR2-mCherry expressing infected cells in superficial layers. C, Light-evoked PSCs at different membrane potentials, allowing discrimination of IPSCs (at 0 mv) and EPSCs (at -80 mV, upper trace). Application of TTX (1 µM) and 4-AP (200 µM) to isolate monosynaptic PSCs abolished IPSCs, but not EPSCs. D, Gabazine application (10 µM) abolishes the IPSC, but not the EPSC (n = 5). E, Quantification of the effects of combined application of TTX and 4-AP (n = 5) on EPSC and IPSC peak amplitudes. F, Dependence of inhibitory and excitatory conductances on the laser power. Recordings in this figure were performed at a laser power eliciting maximal PSC amplitudes. Error bars denote SEM. Download Figure 4-2, TIF file. [file eneuro-12-ENEURO.0065-25.2025-s005.tif]

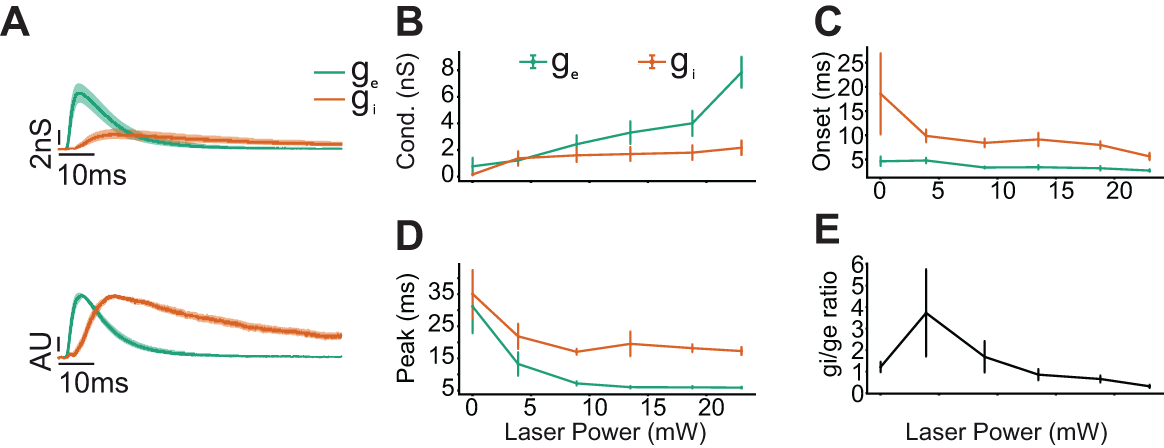

Supplement: Figure 4-3 — Properties of MPP evoked inhibition and excitation in the slice preparation A, Representative excitatory conductance (green) and inhibitory conductance (orange) in response to light stimulation. Inhibitory conductances are smaller and have slower kinetics compared to excitatory conductances. Lower traces normalized to the same peak conductance to illustrate the difference in kinetics. Shaded areas denote SEM. B, Peak excitatory conductance (green) and inhibitory conductance (orange) in response to different light stimulation intensities. Error bars denote SEM. C, Inhibition to excitation ratio for different light stimulation intensities. Error bars denote SEM. D, Time to peak of excitatory conductance (green) and inhibitory conductance (orange) in response to different light stimulation intensities. Error bars denote SEM. E, Time from stimulation to response onset of excitatory conductance (green) and inhibitory conductance (orange) in response different light stimulation intensities. Error bars denote SEM. Download Figure 4-3, TIF file. [file eneuro-12-ENEURO.0065-25.2025-s006.tif]

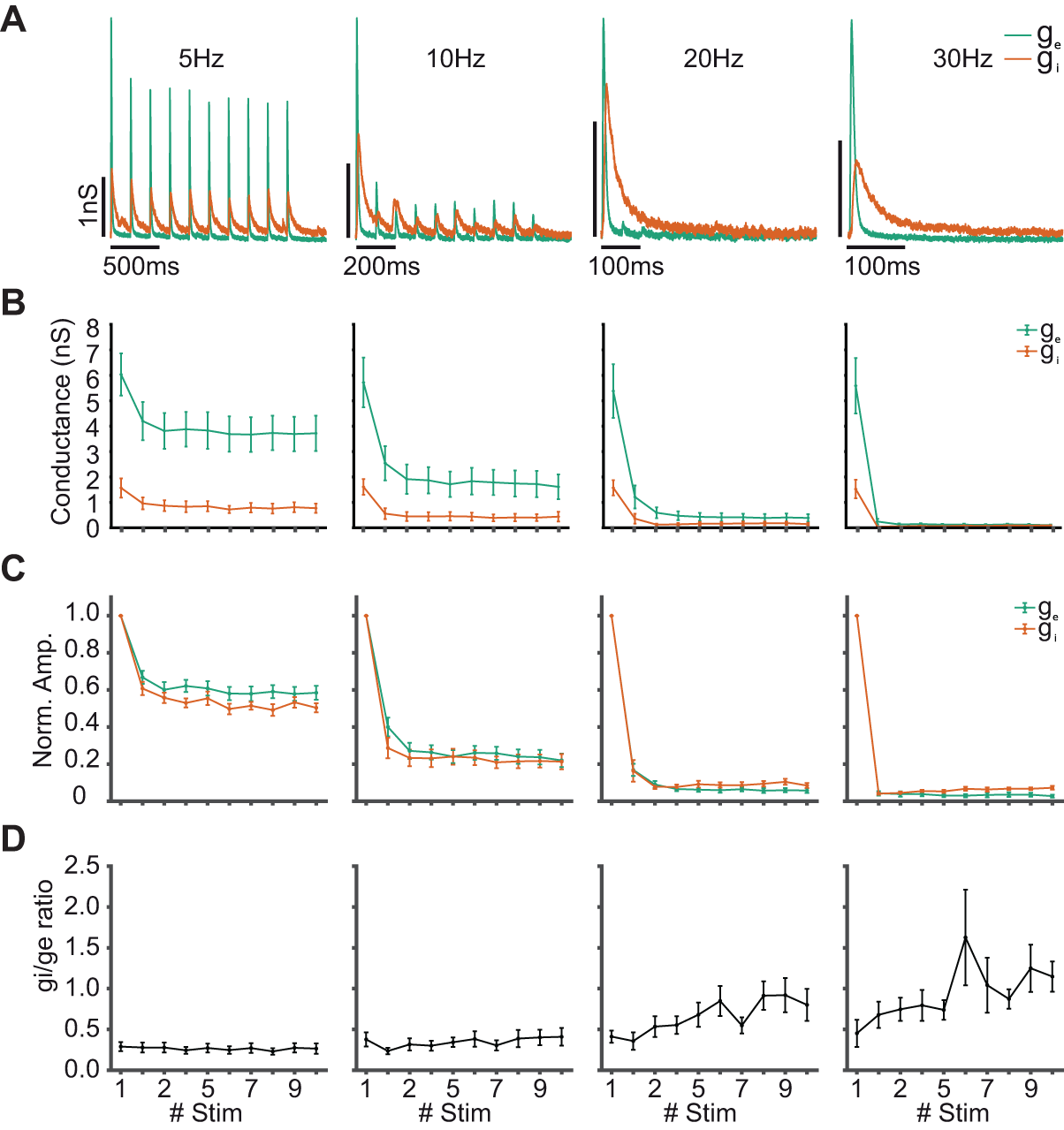

Supplement: Figure 5-1 — Excitation-inhibition balance in granule cells during repetitive stimulation of perforant path in the slice preparation. A, Time courses of excitatory conductance (green) and inhibitory conductance (orange) for 5 Hz, 10 Hz, 20 Hz and 30 Hz stimulation trains. B, Mean conductance amplitudes across granule cells. Error bars denote SEM. Friedman tests for the changes of amplitudes: 5 Hz: n = 11, df = 9, ge: χ2 = 54, p < 0.0001; gi: χ2 = 45, p < 0.0001; 10 Hz: n = 12, df = 9, ge: χ2 = 67, p < 0.0001; gi: χ2 = 40, p < 0.0001; 20 Hz: n = 12, df = 9, ge: χ2 = 61, p < 0.0001 ; gi: χ2 = 38, p < 0.0001; 30 Hz: n = 11, df = 9, ge: χ2 = 35, p < 0.0001; gi: χ2 = 52, p < 0.0001. C, Like B with amplitudes normalized to the peak amplitude of the first PSC in the train. Error bars denote SEM. D, Inhibition to excitation ratio for different stimulation frequencies. Error bars denote SEM. Friedman test, 5 Hz: χ2 = 15, n = 11, df = 9, p = 0.09; 10 Hz: χ2 = 11, n = 12, df = 9, p = 0.25; 20 Hz: χ2 = 37, n = 12, df = 9, p < 0.0001; 30 Hz: χ2 = 31, n = 11, df = 9, p < 0.001. Download Figure 5-1, TIF file. [file eneuro-12-ENEURO.0065-25.2025-s007.tif]
