# Supplementary material for: In Vivo Analysis of Medial Perforant Path-Evoked Excitation and Inhibition in Dentate Granule Cells
Source: eNeuro. 2025 Dec 9;12(12):ENEURO.0065-25.2025. doi: 10.1523/ENEURO.0065-25.2025 (PMC12697386; doi:10.1523/ENEURO.0065-25.2025)
Supplement: Figure 4-4 — Granule cell E-I ratios in the literature, E-I ratios as found in different publications (Citation), the method used, the technique used to isolate inhibitory and excitatory currents/potentials/conductances, the estimated inhibition-excitation ratio and the frequency dependence of the ratio. Download Figure 4-4, DOCX file. [file eneuro-12-ENEURO.0065-25.2025-s008.docx]

| **Citation** | **Method** | **Isolation of IPSCs/EPSCs** | **Estimation of inhibition-excitation ratios** | **Frequency-dependence of inhibition-excitation ratios** |
| --- | --- | --- | --- | --- |
| Ewell and Jones, 2010 ^1^, Fig. 4 | - Measured IPSC/EPSC.  - Electrical stimulation of MML.  - Transverse (axial) brain slice, likely ventral HC. | - No pharmacological dissection  - Neurons held roughly at midpoint between E and I reversal potentials.  - DNQX experiment ruled out direct stimulation of PP fibers. | Maximal ratio observed ~ 3 | Not tested |
| Marin-Burgin… Schinder, 2013 ^2^, Fig. S8 | - Measured IPSC/EPSC.  - Electrical stimulation of MML.  - Coronal brain slices, likely dorsal HC.  - Likely 30°C | - No pharmacological dissection  - Neurons held at -70 and 0 mV for EPSCs and IPSCs, respectively. | 350 pA IPSC, 200 pA EPSC, ratio of ~1.75 | 10 Hz, 10 pulses, EPSCs decrease by ~55%, IPSCs by 40%, implies that Gi/Ge ratios increase. |
| Belén Pardi…. Marin-Burgin, 2015 ^3^, Fig. 3, Supplement 2 | - Measured IPSG/EPSG.  - Electrical stimulation of MML.  - Transverse (axial) brain slice, likely ventral HC.  - Likely 30°C | No pharmacological dissection.  - Neurons held at -60 and 0 mV for EPSCs and IPSCs, respectively. | Ratio around or slightly greater than 2 | Not analysed (did not analyse peak PSCs). But E/I ratios are reduced during 20 Hz trains. Implies that Gi/Ge ratios increase. |
| Hsu et al., 2015 ^4^, Fig. 2, Supp. Fig. S5 | - Measured IPSG/EPSG.  - Optogenetic Stimulation of MPP.  - Coronal brain slices, likely dorsal HC.  - Either room temperature or 34° | No pharmacological dissection.  - Neurons held at -25 and 10 mV for EPSCs and IPSCs, respectively. | Ratio around 2 at room temperature, but at physiological temperature 0.4 (Supp. Fig. S5) | 10 Hz, 10 pulses, Gi/Ge ratios decrease (room temperature). |
| Pofahl et al., (this paper), in-vitro experiments | - Measured IPSG/EPSG.  - Optogenetic Stimulation of MPP.  - Coronal brain slices, dorsal HC.  - Room temperature | - Pharmacological dissection in every measurement.  - Neurons held at -80 and 0 mV for EPSCs and IPSCs, respectively. | Ratios <0.5 | No change for 5 Hz, but >10 Hz shows an increase in Gi/Ge ratios, consistent with in-vitro but different from our in-vivo results. |
| Pofahl et al., (this paper), in-vivo patch-clamp experiments | - Calculated IPSG/EPSG  - Optogenetic Stimulation of MPP.  - Dorsal HC. | - No pharmacological dissection.  - Calculation according to Priebe and Ferster, 2005 ^5^ | Ratios of 7.50 to 5.66 | Gi tends to decrease more than Ge (opposite to in-vitro results above), but ratios are not different with 5 and 20 Hz |

References

1. Ewell, L. A. & Jones, M. V. Frequency-tuned distribution of inhibition in the dentate gyrus. *J. Neurosci.* **30,** 12597–12607 (2010).

2. Marín-Burgin, A., Mongiat, L. A., Pardi, M. B. & Schinder, A. F. Unique processing during a period of high excitation/inhibition balance in adult-born neurons. *Science* **335,** 1238–1242; 10.1126/science.1214956 (2012).

3. Pardi, M. B., Ogando, M. B., Schinder, A. F. & Marin-Burgin, A. Differential inhibition onto developing and mature granule cells generates high-frequency filters with variable gain. *eLife* **4,** e08764; 10.7554/eLife.08764 (2015).

4. Hsu, T.-T., Lee, C.-T., Tai, M.-H. & Lien, C.-C. Differential Recruitment of Dentate Gyrus Interneuron Types by Commissural Versus Perforant Pathways. *Cerebral cortex (New York, N.Y. : 1991)* **26,** 2715–2727; 10.1093/cercor/bhv127 (2016).

5. Priebe, N. J. & Ferster, D. Direction selectivity of excitation and inhibition in simple cells of the cat primary visual cortex. *Neuron* **45,** 133–145 (2005).
